# Genomic and evolutionary insights into a novel Neisseria meningitidis genogroup W clonal complex 11 variant with dual resistance to penicillin and azithromycin in Spain

**DOI:** 10.1099/mgen.0.001703

**Published:** 2026-05-12

**Authors:** Josep Roca-Grande, Albert Moreno-Mingorance, Patricia Álvarez-López, Maider Arando, Alba Bellés-Bellés, Jorge Calvo-Montes, Jordi Càmara, Emilia Cercenado, Vicente Descalzo, M. Ángeles Galán-Ladero, Jorge Néstor García-Pérez, Frederic Gómez, Yannick Hoyos-Mallecot, Joan López-Madueño, Mayli Lung, Andrea Martín-Nalda, Alba Mir-Cros, Carmen Muñoz-Almagro, Daniel Navarro de la Cruz, Inés Oliveira-Souto, M. Ángeles Orellana, Begoña Palop, Amaresh Pérez-Argüello, Mar Olga Pérez-Moreno, Guillem Puigsech-Boixeda, M. Dolores Quesada, Alba Rivera, Carlos Rodrigo, Ana Rodriguez-Fernandez, Enrique Ruiz de Gopegui, Carolina Sarvisé, Núria Serre-Delcor, Pere Soler-Palacín, Aleix Soler-Garcia, Jesús Trejo-Zahínos, Belén Viñado, M. Nieves Larrosa, Juan José González-López

**Affiliations:** 1 Microbiology Research Group, Institut de Recerca Vall d’Hebron (VHIR), Barcelona, Spain; 2 Departament de Genètica i Microbiologia, Universitat Autònoma de Barcelona (UAB), Bellaterra, Spain; 3 CIBER de Enfermedades Infecciosas (CIBERINFEC), Instituto de Salud Carlos III, Madrid, Spain; 4 Infectious Diseases, STI/HIV Unit, Hospital Universitari Vall d'Hebron, Barcelona, Spain; 5 Department of Clinical Microbiology, Hospital Universitari Arnau de Vilanova, Institut de Recerca Biomèdica de Lleida – Fundació Dr. Pifarré, IRBLleida, Av. Alcalde Rovira Roure, 80, 25198, Lleida, Spain; 6 Department of Clinical Microbiology, Hospital Universitario Marqués de Valdecilla-IDIVAL, Santander, Spain; 7 Department of Clinical Microbiology, Hospital Universitari de Bellvitge, IDIBELL-UB, L’Hospitalet de Llobregat, Barcelona, Spain; 8 CIBER de Enfermedades Respiratorias (CIBERES), Instituto de Salud Carlos III, Madrid, Spain; 9 Department of Clinical Microbiology and Infectious Disease, Hospital General Universitario Gregorio Marañón, Madrid, Spain; 10 Department of Clinical Microbiology, Hospital Universitario Reina Sofía, Córdoba, Spain; 11 Department of Clinical Microbiology, Hospital Universitari de Tarragona Joan XXIII, Pere Virgili Health Research Institute (IISPV), Tarragona, Spain; 12 Department of Clinical Microbiology, Hospital Universitari Vall d’Hebron (HUVH), Barcelona, Spain; 13 Fundació Althaia, Xarxa Assistencial Universitària Manresa, Manresa, Spain; 14 Pediatric Infectious Diseases and Immunodeficiencies Unit, Children’s Hospital, Hospital Universitari Vall d’Hebron Campus, Barcelona, Spain; 15 Infectious Diseases and Microbiome Research Group, Institut de Recerca Sant Joan de Déu, Hospital Sant Joan de Déu, Esplugues, Spain; 16 CIBER de Epidemiología y Salud Pública (CIBERESP), Instituto de Salud Carlos III, Madrid, Spain; 17 School of Medicine and Health Sciences, Universitat Internacional de Catalunya (UIC), Barcelona, Spain; 18 Department of Clinical Microbiology, Complejo Hospitalario Universitario de Santiago, Santiago de Compostela, Spain; 19 Tropical Medicine Unit Vall d'Hebron-Drassanes, Infectious Diseases Department, Vall d'Hebron University Hospital, PROSICS Barcelona, Barcelona, Spain; 20 Department of Clinical Microbiology, Hospital Universitario 12 de Octubre, Madrid, Spain; 21 Department of Clinical Microbiology, Hospital Regional Universitario de Málaga, Málaga, Spain; 22 Department of Clinical Microbiology, Hospital Universitari de Tortosa Verge de la Cinta, Tortosa, Spain; 23 Department of Clinical Microbiology, Hospital Universitari Germans Trias i Pujol, UAB, Badalona, Spain; 24 Department of Clinical Microbiology, Hospital de la Santa Creu i Sant Pau, Sant Pau Biomedical Research Institute (IIB Sant Pau), Barcelona, Spain; 25 Department of Paediatrics, Hospital Universitari Germans Trias i Pujol, Universitat Autònoma de Barcelona (UAB), Badalona, Spain; 26 Department of Clinical Microbiology, Hospital Universitario Son Espases, Instituto de Investigación Sanitaria Illes Balears (IdISBa), Palma de Mallorca, Spain; 27 Department of Pediatrics, Hospital Sant Joan de Déu, Esplugues, Spain

**Keywords:** antimicrobial resistance, cc11, Bayesian phylogenetics, molecular epidemiology, *Neisseria meningitidis*, Spain

## Abstract

*Neisseria meningitidis* clonal complex 11 (cc11) is a hyperinvasive lineage associated with global dissemination and outbreaks, including the ‘South American/UK’ sublineage. In Spain, cc11 serogroup W cases have increased in recent years; however, the evolutionary dynamics of circulating variants remain insufficiently characterized. This study aimed to assess the clonal diversity, temporal evolution and antimicrobial resistance profiles of cc11 isolates recovered in Spain between 2011 and 2025. A total of 1,226 isolates (406 invasive and 820 non-invasive) were collected across 8 Spanish regions, from which cc11 isolates were selected for analysis. Whole-genome sequencing (WGS) was used to perform core-genome multilocus sequence typing and SNP-based Bayesian phylogenetics to characterize population structure. Antimicrobial susceptibility was assessed using gradient diffusion, and genetic determinants of resistance were identified by WGS. We identified 179 cc11 isolates, mostly belonging to genogroup W (73.7%) and lineage 11.1 (85.5%). The ‘South American/UK’ sublineage was the main sublineage and displayed extensive diversification. A previously undescribed variant, designated the ‘2020 strain’, emerged with an estimated most recent common ancestor in 2017 and accounted for 77.2% of ‘South American/UK’ sublineage isolates recovered between 2023 and 2025. This variant was mainly identified in respiratory samples from men who have sex with men but was also associated with invasive meningococcal disease in other population groups. The ‘2020 strain’ exhibited resistance to penicillin and azithromycin, likely driven by penicillin-binding protein 2 and MtrCDE alterations. The emergence of this antimicrobial-resistant variant highlights the adaptive capacity of cc11 and the importance of genomic surveillance to monitor its evolution and public health relevance.

**Impact Statement**

*Neisseria meningitidis* clonal complex 11 (cc11) is one of the most hypervirulent and rapidly evolving meningococcal populations circulating globally. By integrating high-resolution genomics with detailed epidemiological data, our study provides a comprehensive characterisation of the cc11 isolates circulating in Spain over the past 15 years. Our results reveal a remarkable genetic variability within this clonal complex, including the identification of a previously undescribed cc11 variant that has recently expanded, showing clear evidence of evolutionary diversification and acquisition of antimicrobial resistance determinants not previously documented within the cc11 population. This emerging variant illustrates the ongoing evolution of meningococcal populations and reinforces the importance of sustained genomic surveillance to identify the emergence and spread of antimicrobial resistance and other features of epidemiological relevance.

## Data Summary

The genome sequence reads of the 179 *Neisseria meningitidis* cc11 isolates analysed in this study were deposited in the National Center for Biotechnology Information database under BioProject accession no. PRJNA1268441. The genome assembly sequences of the isolates used in this study were deposited in the PubMLST *Neisseria* spp. database. The corresponding accession numbers, BioSample and PubMLST identifiers, along with relevant metadata, are provided in Table S1.

## Introduction

*Neisseria meningitidis* (*Nm*) is the causative agent of invasive meningococcal disease (IMD) and can be classified into 12 capsular serogroups, of which six (A, B, C, W, X and Y) are responsible for most global IMD cases [1]. The use of genomic tools, particularly whole-genome sequencing (WGS), has significantly improved the resolution of meningococcal typing in recent years. WGS provides valuable information regarding the diversity and dynamics of meningococcal populations, mainly through (i) genotyping of the capsule locus to infer serogroup; (ii) multilocus sequence typing (MLST), based on seven housekeeping genes that define a sequence type (ST); and (iii) the identification of clonal complexes (cc), which group genetically related STs [2].

In Spain, serogroup B has been the most prevalent among IMD cases since the early 2000s. However, the incidence of serogroup W has increased since 2017 and is currently established as the second most prevalent serogroup [3], particularly associated with the hyperinvasive lineage belonging to cc11 [4]. Phylogenomic analyses of cc11 isolates revealed a highly complex population structure, characterized by extensive genetic diversity [5]. Historically, cc11 isolates have been mainly associated with serogroups B and C, usually linked to lineage 11.2 or proximal regions of lineage 11.1, while serogroup W isolates belonging to cc11 are linked to distal regions of lineage 11.1 [6]. In the early 2000s, serogroup W cc11 emerged as the cause of an epidemic during the Hajj pilgrimage in Mecca (Saudi Arabia), subsequently spreading to close contacts of the returning pilgrims. This outbreak strain later disseminated globally and became the predominant cc11 strain among serogroup W isolates during the first decade of the 2000s [7,8]. However, from the 2010s onwards, a new wave of IMD cases related to serogroup W was observed in Europe [9], which was not related to the ‘African/Hajj outbreak’ sublineage, but rather to a new sublineage imported from South America and named the ‘South American/UK’ sublineage [6,10].

The ‘South American/UK’ sublineage was first reported in Brazil, Argentina and Chile, leading to the ‘South American’ variant [5,11, 12], which later spread to Europe and was initially identified in the UK. There, it gave rise to a diversification known as the ‘original UK’ variant, which further evolved into the ‘2013 novel strain’ variant [10,13,15]. In 2016, a new variant including isolates with resistance or reduced susceptibility to penicillin, referred to as the ‘2015 strain’, was identified in Australia and New Zealand. These isolates, characterized by the presence of the *penA* allele 9 (*penA*9), derived from the ‘2013 novel strain’ [16,17].

Traditional typing methods, such as sequencing of outer membrane proteins (e.g. PorA, PorB and FetA) or MLST, usually lack the resolution required to distinguish between 11.1 sublineages and variants [6]. Therefore, WGS is currently considered the most robust and informative approach to investigate the genetic relationships among cc11 isolates, enabling the identification of links between contemporary and historical cases, as well as the origin and spread of emerging strains.

This study aimed to unravel the clonal diversity and evolutionary dynamics of *Nm* cc11 isolates recovered in Spain between 2011 and 2025, including the identification of a novel variant within the ‘South American/UK’ sublineage linked to penicillin and azithromycin resistance mediated by variations in the penicillin-binding protein 2 (PBP2) and the MtrCDE efflux pump, respectively.

## Methods

### Isolates and sociodemographic data collection

We compiled a large collection of *Nm* isolates from 57 Clinical Microbiology Laboratories across eight Spanish regions (Catalonia, Community of Madrid, Cantabria, Andalusia, Balearic Islands, Galicia, Aragon and Canary Islands) between January 2011 and June 2025. The isolates were obtained from patients attending hospitals, primary healthcare centres and sexually transmitted infection (STI) screening clinics, following the routine diagnostic procedures.

The collection included 1,226 isolates obtained from the following sample types: 473 respiratory samples collected during STI screening (throat swabs), 406 sterile sites/fluids (blood, cerebrospinal fluid and others), 298 respiratory samples not associated with STI screening (throat swabs, sputum, bronchial aspirate, bronchoalveolar lavage and tracheal aspirate) and 49 urogenital/anorectal samples (rectal, urethral, vaginal and cervical swabs, as well as semen and urine samples). The distribution of isolate types varied over time. As shown in Fig. S1 (available in the online Supplementary Material), isolates from respiratory samples collected during STI screening and from urogenital/anorectal sites were predominantly obtained between 2023 and 2025, providing enhanced representation of these specimen types during the most recent years of the study.

### WGS and bioinformatic analysis

Isolates were cultured in PVX or VCA3 agar plates (bioMérieux, Marcy-l’Étoile, France) for 18–24 h at 37 °C in a 5% CO_2_ atmosphere. Short-read sequencing was performed for all 1,226 isolates. DNA was extracted using the DNeasy UltraClean Microbial Kit (QIAGEN, Hilden, Germany). Libraries were prepared using the DNA Prep Kit and sequenced using the MiSeq and/or NextSeq 2000 devices (Illumina, San Diego, USA) according to the manufacturer’s instructions. Trimmomatic (v.039), Unicycler (v0.4.8) and SPAdes (v3.14.1) were used for raw-read trimming and *de novo* genome assembly. Assemblies were submitted to the *Neisseria* spp. PubMLST database (https://pubmlst.org/organisms/neisseria-spp/) to characterize capsular genogroup, ST and cc. Among the 1,226 sequenced isolates, those assigned to cc11 by PubMLST were selected for the present study. The temporal distribution of the cc11 isolates is also shown in Fig. S1. Additionally, *penA*, NEIS3240(*bla*_ROB-1_), NEIS2357(*bla*_TEM-1_), *gyrA*, *parC*, NEIS1632 (*mtrE*), NEIS1633(*mtrD*), NEIS1634(*mtrC*), NEIS1635(*mtrR*) and pro_NEIS1635 alleles and information regarding the presence of nucleotide and amino acid substitutions linked to antimicrobial resistance were identified according to the PubMLST locus definition [18]. To study clonal diversity, genomes were compared with the ‘Genome Comparator’ analysis tool available in PubMLST, using *N. meningitidis* core-genome multilocus sequence typing (*N. meningitidis* cgMLST) v3.0 that includes 1,329 core loci. The resulting distance matrix was evaluated with SplitsTree v4.19.0 [19] to generate a neighbor-net phylogenetic network. A panel of 12 reference cc11 genomes representing the known diversity of cc11 was included for contextualization (Table S2).

A dated phylogenetic analysis was performed to investigate the evolutionary dynamics of our cc11 isolate collection (*n*=179) together with previously described cc11 reference strains (*n*=12). Raw sequence data (FASTQ files) for the reference strains were retrieved from the National Center for Biotechnology Information database (Table S2). These FASTQ files were also filtered using Trimmomatic v0.39, and the resulting reads were mapped against the complete genome of *Nm* strain 16–579 (serogroup W, ST-11, cc11; GenBank accession no. CP030814.1) using Snippy v.4.3.6 to identify SNPs and generate a SNP alignment. Recombination regions were identified and removed using Gubbins v2.4.1 [20]. The recombination-filtered SNP tree generated by Gubbins, along with the sampling date for each isolate (or the year range when the sampling date was not available), was used to perform a root-to-tip regression analysis to evaluate the temporal signal (Fig. S2) and to construct a Bayesian dated phylogeny using BactDating v1.1.2. Four molecular clock models [Poisson, negative binomial, mixed gamma and mixed continuous additive relaxed clock (cARC)] were performed with a total of 10^7^ iterations each to ensure that the effective sample size of the inferred parameters exceeded 200 in the Markov chain Monte Carlo analysis. The model with the lowest deviance information criterion (DIC) was used for the final analysis. To further assess the robustness of the temporal signal, the analysis was repeated after forcing all sampling dates to be equal under the same conditions, confirming that the observed signal was not explained by the phylogenetic structure alone. The estimated date of the most recent common ancestor (MRCA) and its 95% highest posterior density (HPD) were obtained directly from the BactDating output, inferred based on genetic divergence and sampling dates [21].

### Antimicrobial susceptibility testing

Antimicrobial susceptibility was assessed using the gradient diffusion method (Etest^™^, bioMérieux) for penicillin G, ceftriaxone, cefotaxime, meropenem, ciprofloxacin, azithromycin and rifampicin. The isolates were incubated for 18–24 h at 37 °C in a 5% CO_2_ atmosphere on Mueller–Hinton agar supplemented with 5% horse blood and 20 mg l^−1^ *β*-NAD (bioMérieux). Minimum inhibitory concentrations (MICs) were interpreted according to the European Committee on Antimicrobial Susceptibility Testing (EUCAST) 2025 clinical breakpoint values (https://eucast.org/), except for azithromycin, for which the Clinical & Laboratory Standards Institute (CLSI) 2025 clinical breakpoint values were applied (https://em100.edaptivedocs.net/).

## Results

### Bacterial isolates and patients’ demographics

Among the 1,226 *Nm* isolates collected across Spain between January 2011 and June 2025, 179 cc11 meningococcal isolates were obtained from 179 distinct patients. Among these, 60.3% (108/179) were recovered from invasive samples, 29.6% (53/179) from respiratory samples collected during STI screening, 5.6% (10/179) from respiratory samples not associated with STI screening and 4.5% (8/179) from urogenital/anorectal samples.

The mean age of individuals from whom cc11 isolates were obtained was 42.6±26.2 years, with a median age of 36 years (range: 1 month–94 years old). Regarding sex, 62% (111/179) of the isolates were recovered from male individuals, of whom 52.3% (58/111) were identified as men who have sex with men (MSM). Mean and median age, male proportion and MSM proportion of individuals, stratified by sample type, are shown in Table 1. Notably, respiratory isolates obtained during STI screening and urogenital/anorectal isolates were mainly recovered from MSM, accounting for 96.2% (50/52) and 75% (6/8) of male individuals in each group, respectively (Table 1). A similar MSM distribution according to sample type was observed in the overall collection of 1,226 *Nm* isolates (Fig. S3).

**Table 1.** Mean and median age, male proportion and MSM proportion of individuals from whom samples and *N. meningitidis* isolates belonging to clonal complex 11 were obtained, according to sample type

	Invasive isolates (*n*=108)	Respiratory isolates obtained during STI screening (*n*=53)	Respiratory isolates (*n*=10)	Urogenital/anorectal isolates (*n*=8)	Total (*n*=179)
Mean age, *years±**sd*	46.5±30.8	33±8.3	60.4±24.9	30.6±12.1	42.6±26.2
Median age, *years (range)*	50 (1m–94)	32 (20–56)	69 (12–88)	25 (21–56)	36 (1m–94)
Male proportion, *n* (%)	44 (40.7)	52 (98.1)	7 (70.0)	8 (100.0)	111 (62)
MSM proportion among males, *n* (%)	2 (4.5)	50 (96.2)	0 (0.0)	6 (75.0)	58 (52.3)

Regarding genogroup distribution, 73.7% (132/179) belonged to genogroup W (MenW), 20.1% (36/179) to genogroup C (MenC), 1.1% (2/179) to genogroup B (MenB) and 5% (9/179) were non-groupable (NG). Genogroup distribution according to sampling source is shown in Table S3. The ST most frequently identified was ST-11, accounting for 86% (154/179) of the isolates, followed by ST-18054 (5%; 9/179), ST-10651 (1.7%; 3/179), ST-17488 (1.1%; 2/179) and ST-17612 (1.2%; 2/179). The remaining STs were represented by a single isolate each: ST-1237, ST-2942, ST-10140, ST-11760, ST-14822, ST-14955, ST-14956, ST-15701 and ST-17527.

### Population structure and cgMLST analysis

**Fig. 1.**
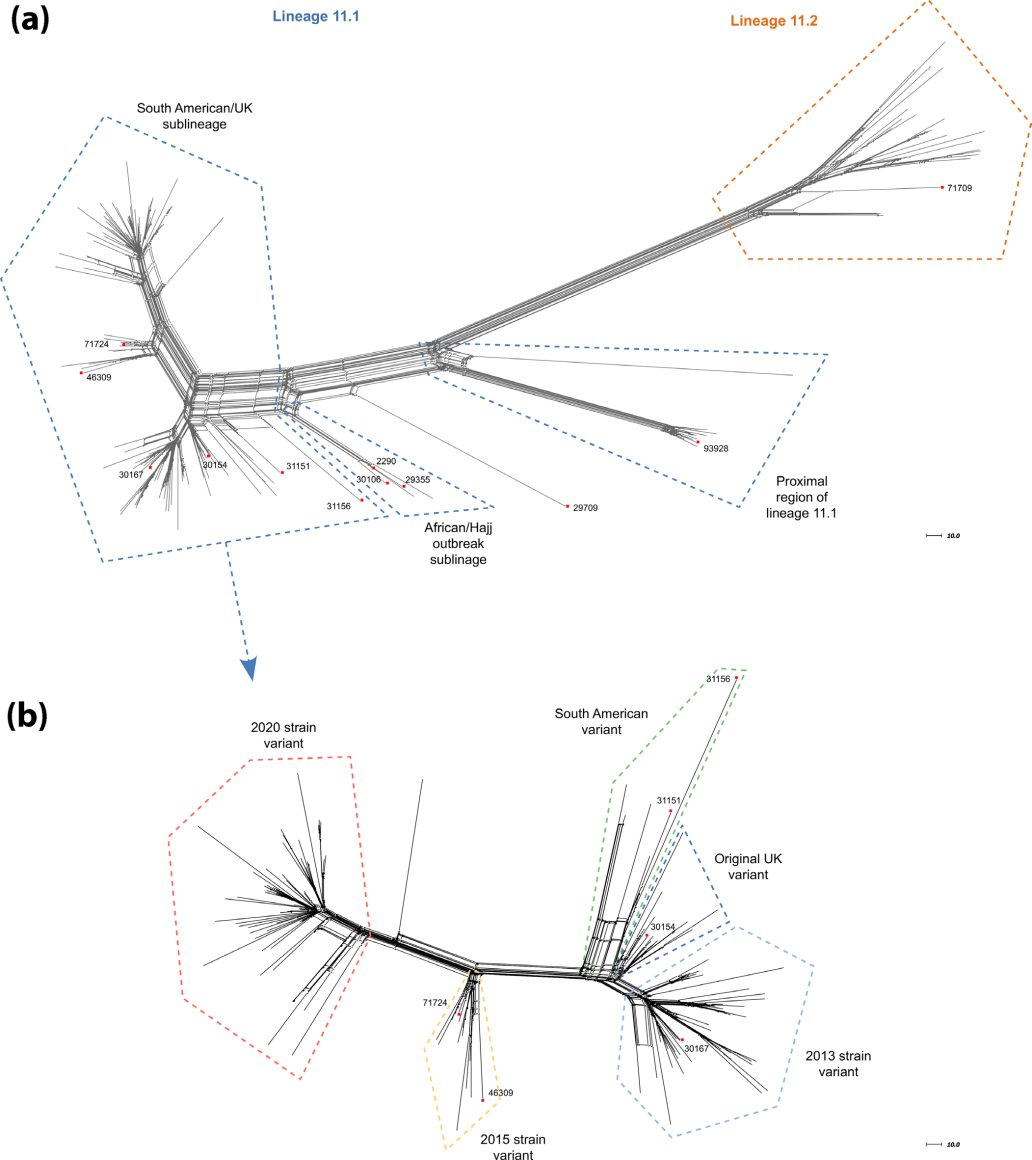
(**a**) Neighbor-net phylogenetic network of 179 *N. meningitidis* cc11 genomes based on allele distances from the *N. meningitidis* cgMLST scheme v3.0 (1,329 loci). Reference genomes (*n*=12) are indicated by red squares, with their corresponding PubMLST identifiers. (**b**) Isolates belonging to the ‘South American/UK’ sublineage underwent a separate *N. meningitidis* cgMLST v3.0 comparison. The scale bar indicates the number of allelic differences among the 1,329 loci compared.

cgMLST analysis revealed a broad genetic diversity, reflected by the presence of several well-defined clusters throughout the neighbor-net phylogenetic network. Overall, 85.5% (153/179) of the cc11 isolates belonged to lineage 11.1, whereas lineage 11.2 accounted for 14.5% (26/179). Lineage 11.1 exhibited a higher diversity than lineage 11.2, comprising isolates assigned to two distinct sublineages and several variants (Fig. 1). Within lineage 11.1, the ‘South American/UK’ sublineage accounted for 90.9% (139/153) of the isolates. The remaining isolates from lineage 11.1 clustered within the proximal region of lineage 11.1 (7.8%, 12/153) or within the ‘African/Hajj outbreak’ sublineage (1.3%, 2/153) (Fig. 1a).

Within the ‘South American/UK’ sublineage, four previously described variants were identified (Fig. 1b): the ‘South American’ variant (4/139), the ‘Original UK’ variant (13/139), the ‘2013 strain’ variant (46/139) and the ‘2015 strain’ variant (12/139). The remaining 64 isolates clustered together and did not group with any previously defined variant. This cluster was designated the ‘2020 strain’ variant, as the earliest isolate was recovered in 2020. However, the most divergent isolate appeared to represent an intermediate between the ‘2015 strain’ and the ‘2020 strain’ variants and was not assigned to either of them, and may represent a recombinant between these two variants.

### Dated phylogenetic analysis of cc11 isolates

To reconstruct the phylogenetic relationships among *Nm* cc11 isolates, a genome-wide SNP-based Bayesian analysis was performed. Root-to-tip regression analysis revealed a significant moderate temporal signal (*R*^2^=0.59, *P*<0.0001), supporting the suitability of the dataset for molecular dating. Comparison of model fits based on the DIC indicated that the cARC model (DIC=2406.17) provided a better fit to the data compared with the other strict or relaxed clock models tested. The same model run with forced equal sampling dates yielded a substantially higher DIC (DIC=4367.25), confirming that the observed temporal signal was not explained by the phylogenetic structure alone. The mean estimated evolutionary rate was 3.65 substitutions/genome/year (95% HPD: 3.17–4.11), corresponding to 2.39×10^−6^ substitutions/site/year.

**Fig. 2.**
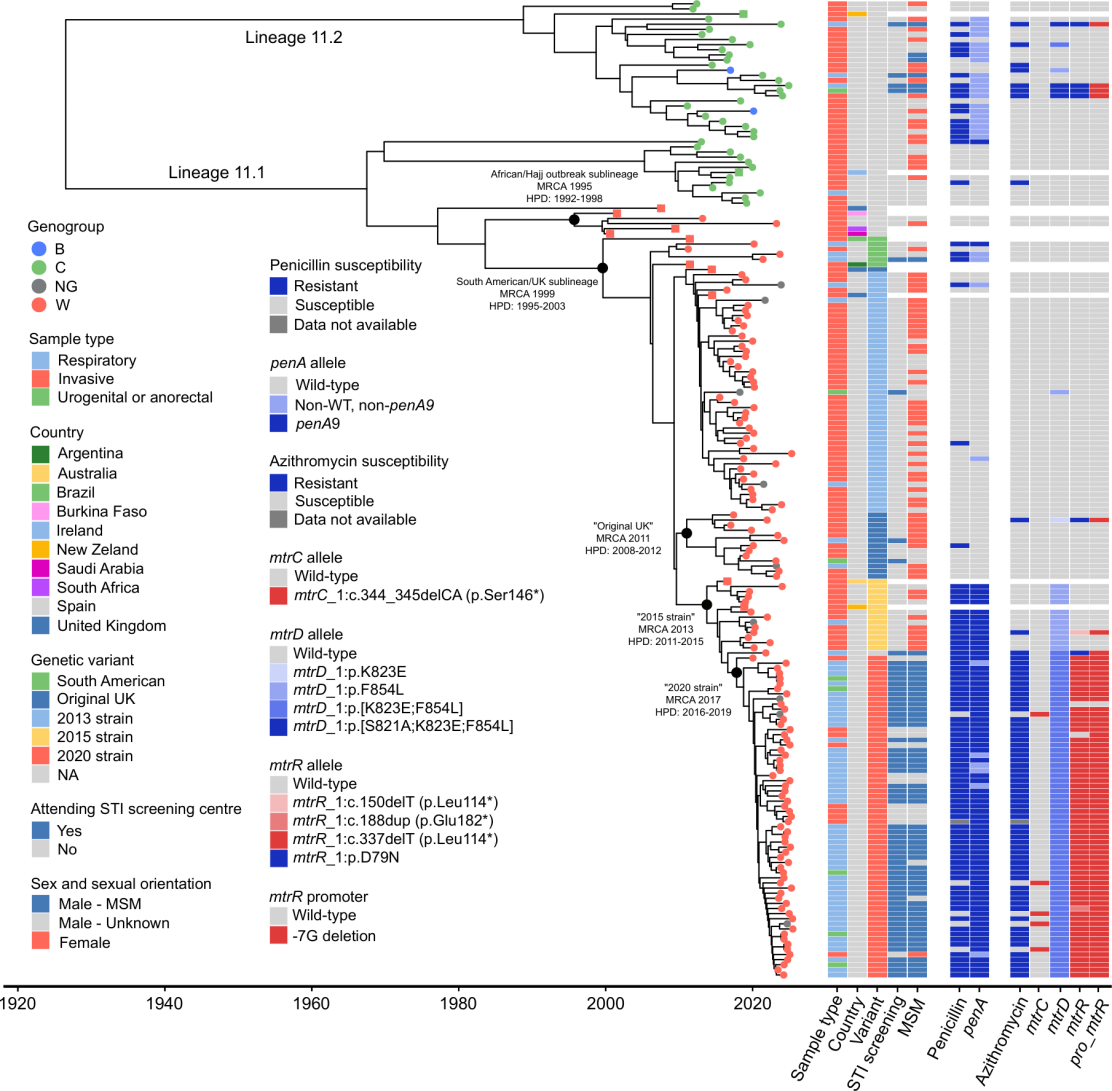
Time-scaled phylogenetic tree of the 179 *N. meningitidis* cc11 genomes identified in this study, along with reference genomes (*n*=12) representing the known genetic diversity of cc11. Coloured dots at the tips of the branches indicate the genogroup. Epidemiological and antimicrobial resistance data for each isolate are shown in the panels on the right. Reference genomes are indicated with coloured squares instead of dots; data regarding STI screening, MSM status and antimicrobial resistance profiles were not available for reference genomes and thus not displayed. NG, non-groupable; NA, not applicable; WT, wild-type.

**Fig. 3.**
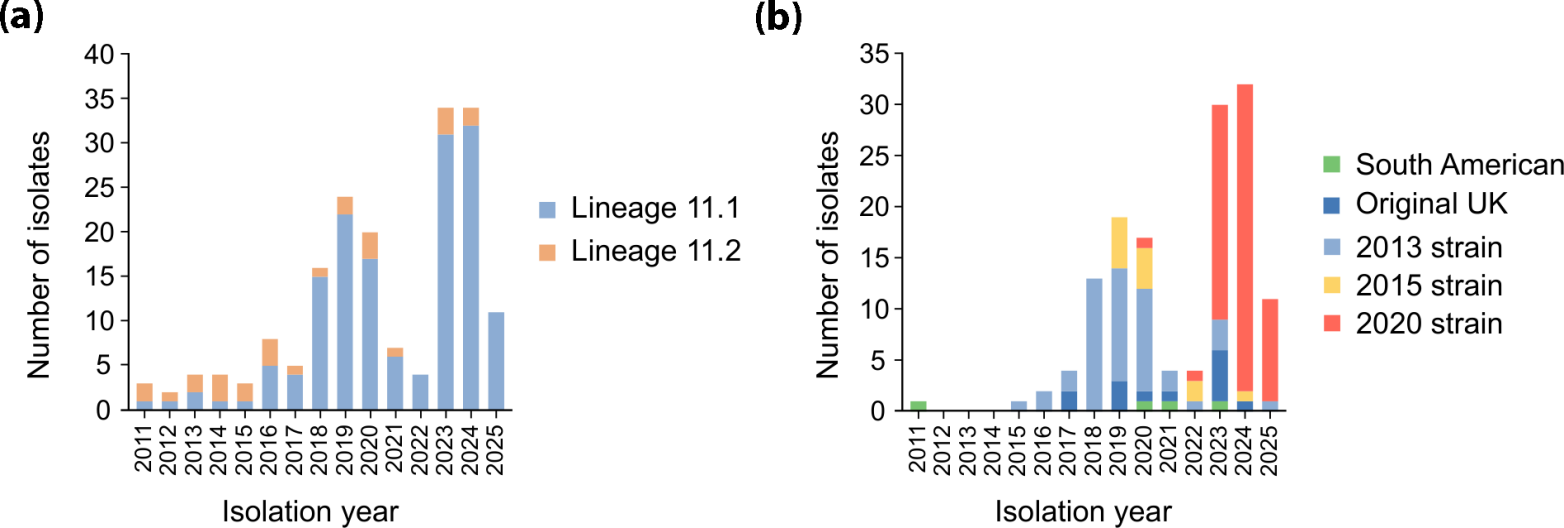
Stacked bar charts showing (**a**) the distribution of cc11 lineages among the 179 *N. meningitidis* cc11 genomes identified in this study and (**b**) the distribution of ‘South American/UK’ sublineage variants among the 139 genomes assigned to this sublineage, stratified by year of isolation.

The phylotemporal analysis (Fig. 2) estimated the time to the MRCA to the year 1926 (95% HPD: 1908–1941), and the pairwise SNP distances ranged from 0 to 432. Lineage 11.2 included the only two MenB isolates identified in this study and 66.7% (24/36) of MenC isolates. Importantly, none of the MenW or NG isolates clustered within this lineage. Isolates belonging to lineage 11.2 were recovered between 2011 and 2024, representing the predominant lineage during 2011–2015 (62.5%, 10/16; Fig. 3a), with 84.6% (22/26) being associated with IMD.

Lineage 11.1 isolates were identified throughout the entire study period and predominated from 2016 to 2025 (90.2%, 147/163, Fig. 3a). This lineage included 33.3% (12/36) of the MenC isolates, which were restricted to the proximal region of lineage 11.1 and gave rise to an early divergent branch in the phylotemporal tree relative to the other isolates within lineage 11.1 (Fig. 2). Except for the most divergent isolate, all members of this branch formed a deep phylogenetic cluster. These isolates were recovered between 2012 and 2021, and all were associated with IMD except for a single respiratory isolate recovered in 2021. Lineage 11.1 also encompassed all the 132 MenW and 9 NG isolates identified in this study. Only two isolates belonged to the ‘African/Hajj outbreak’ sublineage, both associated with IMD cases and recovered in 2013 and 2023, respectively, for which the MRCA was estimated to be in 1995 (HPD: 1992–1998). The remaining 139 isolates belonged to the ‘South American/UK’ sublineage, with an estimated MRCA in 1999 (HPD: 1995–2003).

Phylotemporal reconstruction revealed a scaled population structure within the ‘South American/UK’ sublineage (Fig. 2), corresponding to distinct variants that emerged during successive periods of clonal expansion. The first ‘South American’ variant isolate was recovered from an IMD case in 2011, and the remaining three were obtained from respiratory samples in 2020, 2021 and 2023 (Fig. 3b). Isolates belonging to the ‘2013 strain’ variant were recovered between 2015 and 2025 and represented the majority of ‘South American/UK’ sublineage isolates recovered during the years 2018, 2019 and 2020 (56.7%, 34/60, Fig. 3b). Most were associated with IMD (91.3%; 42/46), and one isolate was from a urethral swab. The 13 isolates belonging to the ‘Original UK’ variant formed a distinct clade within the temporal phylogeny, with an estimated MRCA in 2011 (HPD: 2008–2012). These isolates were recovered between 2017 and 2024, mainly from IMD cases (76.9%, 10/13), and one isolate was from a urethral swab. The MRCA for the ‘2015 strain’ variant was estimated in 2013 (HPD: 2011–2015), while the isolates included in this study were recovered during 2019–2020, and one isolate was detected in 2024 (Fig. 3b). Among these, 91.7% (11/12) were associated with IMD.

The dated phylogenetic analysis estimated the time to the MRCA of the novel ‘2020 strain’ variant identified in this study to be 2017 (95% HPD, 2016–2019). The 63 isolates belonging to this variant showed a close genetic relationship, with pairwise SNP distances ranging from 0 to 39. Of these isolates, 96.8% (61/63) were recovered during 2023–2025 and represented 77.2% (61/79) of the ‘South American/UK’ sublineage isolates obtained during the same period (Fig. 3b). Among the 63 isolates, 77.8% (49/63) were respiratory isolates, 14.3% (9/63) were invasive isolates, while the remaining 7.9% (5/63) were urogenital/anorectal isolates.

All but two of the respiratory isolates belonging to the ‘2020 strain’ variant (95.9%; 47/49) were recovered from male individuals attending an STI screening centre, and 95.7% of these (45/47) were MSM. Similarly, all five urogenital/anorectal isolates from this variant were obtained from MSM. Interestingly, none of the patients who developed IMD could be identified as MSM, nor could they be epidemiologically linked to the MSM community (Fig. 2). Moreover, the mean age of IMD cases was 67.8 years (median: 67; range: 29–91), which was considerably higher than that of individuals carrying respiratory and urogenital/anorectal isolates from this variant, whose mean age was 34.2 years (median: 33; range: 20–66).

### Antimicrobial susceptibility of the ‘2020 strain’ isolates

Antimicrobial susceptibility data were available for 62 of the 63 isolates from the ‘2020 strain’ variant and showed that 91.9% (57/62) of the isolates were resistant to both penicillin (PEN^R^; MIC range: 0.38–0.75 µg ml^−1^) and azithromycin (AZ^R^; MIC range: 3–12 µg ml^−1^), while 3.2% (2/62) were resistant to ciprofloxacin (CIP^R^; MIC: 0.032 µg ml^−1^). Antimicrobial susceptibility data for the remaining antimicrobials, stratified by lineage, sublineage and genetic variants, are shown in Table 2.

**Table 2.** Antimicrobial resistance rates of *N. meningitidis* isolates belonging to clonal complex 11 obtained in this study from 2011 to 2025, classified according to lineage, sublineage and genetic variant

	Lineage 11.2 (*n*=26), *n* (%)	Proximal region 11.1 (*n*=12), *n* (%)	African/Hajj outbreak (*n*=2), *n* (%)	South American (*n*=4), *n* (%)	Original UK (*n*=13), *n* (%)	2013 strain (*n*=46), *n* (%)	2015 strain (*n*=12), *n* (%)	2020 strain (*n*=62), *n* (%)	Total (*n*=178), *n* (%)
Antimicrobial agent									
Penicillin G	15 (57.7)	2 (16.7)	0 (0)	3 (75)	1 (7.7)	2 (4.4)	12 (100)	57 (91.9)	93 (52.3)
Cefotaxime	0 (0)	0 (0)	0 (0)	0 (0)	0 (0)	0 (0)	0 (0)	0 (0)	0 (0)
Ceftriaxone	0 (0)	0 (0)	0 (0)	0 (0)	0 (0)	0 (0)	0 (0)	0 (0)	0 (0)
Meropenem	0 (0)	0 (0)	0 (0)	0 (0)	0 (0)	0 (0)	0 (0)	0 (0)	0 (0)
Azithromycin	7 (26.9)	1 (8.3)	0 (0)	0 (0)	1 (7.7)	0 (0)	1 (8.3)	57 (91.9)	68 (38.2)
Ciprofloxacin	0 (0)	0 (0)	0 (0)	0 (0)	0 (0)	0 (0)	0 (0)	2 (3.2)	2 (1.1)
Rifampicin	0 (0)	0 (0)	0 (0)	0 (0)	0 (0)	0 (0)	0 (0)	0 (0)	0 (0)

Analysis of antimicrobial resistance determinants revealed that PEN^R^ isolates from the ‘2020 strain’ variant encoded alleles *penA*9 (89.5%; 51/57), *penA*11 (1/57), *penA*12 (1/57), *penA*20 (1/57), *penA*271 (1/57) and *penA*1216 (2/57), with all encoding the five typical amino acid substitutions (F504L, A510V, I515V, H541N and I566V) in PBP2 associated with PEN^R^ [22,23]. No *β*-lactamase encoding gene was identified in any isolate of the study.

Genetic analysis of AZ^R^ isolates belonging to the ‘2020 strain’ variant revealed that all the isolates harboured the K823E and F854L amino acid substitutions in MtrD, associated with decreased susceptibility to azithromycin in *Neisseria gonorrhoeae* (*Ng*) [24,25]. The most frequent identified alleles were *mtrD*4108 (54/57), *mtrD*5624 (1/57), *mtrD*5630 (1/57) and *mtrD*6258 (1/57). Regarding *mtrR*, 98.2% (56/57) carried a −7G deletion in the *mtrR* promoter region, and 96.5% (55/57) encoded a frameshift mutation in *mtrR* that resulted in a premature stop codon. Of these, 54 isolates harboured the mtrR_1:c.337delT (p.Leu114*) mutation (newly identified allele *mtrR*3151), whereas one isolate carried the mtrR_1:c.188dup (p.Glu182*) mutation (newly identified allele *mtrR*3149). In contrast, the single AZ^R^ isolate that lacked mutations in *mtrR* or its promoter exhibited a distinct *mtrE* allele (*mtrE*2477), which displayed several amino acid mutations (E252D, T314S, G316D, G318H, V355A and S369A) relative to *mtrE*1, the allele found in all the other isolates. These substitutions could potentially contribute to the AZ^R^ phenotype. Regarding the CIP^R^ isolates, no mutations in *gyrA* (allele *gyrA*4) and *parC* (allele *parC*44) that could be associated with resistance to this antimicrobial were identified.

Remarkably, five isolates remained susceptible to penicillin (MIC range: 0.094–0.125 µg ml^−1^) and azithromycin (0.064–0.125 µg ml^−1^) despite presenting the *penA*9 allele and both the *mtrD* and *mtrR* mutations described above. All of these isolates harboured an identical frameshift mutation within a GC dinucleotide hexarepeat region in the coding sequence of *mtrC*, which resulted in the presence of a premature stop codon [mtrC_1:c.344_345delCA (p.Ser146*)], corresponding to the newly identified allele *mtrC*2948, likely impairing correct translation and expression of MtrC.

## Discussion

The epidemiology of *Nm* cc11 isolates in Spain has undergone substantial changes, reflecting the circulation of diverse lineages and genetic variants described worldwide, as well as the emergence of a novel variant. Our results indicate that MenC isolates belonging to lineage 11.2 and the proximal region of lineage 11.1 were the main circulating strains detected between 2011 and 2015. In contrast, between 2016 and 2025, most isolates belonged to MenW and the ‘South American/UK’ sublineage, mainly associated with recent genetic variants, such as the ‘2013 strain’, the ‘2015 strain’ and the novel ‘2020 strain’ variants. However, the *Nm* isolates included in this study do not represent all isolates obtained from patients in the Spanish regions covered during the study period. In particular, our study lacks representative respiratory and urogenital/anorectal isolates data from the early years of the study period, which limits our ability to determine whether the apparent increase in cc11 detection reflects an increase or an improved detection due to enhanced surveillance of genetic variants circulating in these sites. Despite these limitations, our comprehensive analysis provides a robust overview of the evolutionary trajectories, strain replacement patterns and the emergence of novel variants within cc11 in Spain.

The ‘2020 strain’ variant identified in this study was defined by (i) its genetic identity, (ii) its divergence from the ‘2015 strain’ and the other genetic variants based on cgMLST allele distances and (iii) the acquisition of azithromycin resistance. This variant was mainly identified among individuals within the MSM community and was identified in respiratory, urogenital and anorectal sites. The widespread use of azithromycin for treating various STIs during the past decade has exerted strong selective pressure, contributing to AZ^R^ in several STI pathogens [26,27]. This selective pressure has likely also affected *Nm*, promoting the successful expansion of this novel AZ^R^ variant, as has been previously described for other pathogens circulating in MSM populations, such as *Shigella* spp. or *Haemophilus parainfluenzae* [28,29]. Notably, no IMD cases related to this variant could be identified in MSM individuals during the study period. Overall, these findings may indicate clonal transmission of this emergent variant within MSM networks. However, this interpretation is limited by the intensive targeted screening performed in MSM populations, which excludes assessment of whether this emergent variant is also circulating in other population groups, such as men who have sex with women, adolescents or university students. Consequently, additional studies investigating carriage in populations outside the MSM community are warranted to determine whether circulation is restricted to these networks or is more widespread.

Previous studies in populations with elevated carriage risk, such as adolescents and MSM groups, generally report a low prevalence of MenW:cc11 [30,34]. Nevertheless, the circulation and transmission of MenW:cc11 have occasionally been documented. For example, increased MenW:cc11 carriage was reported among university students in England, largely associated with the ‘2013 strain’ variant and facilitated by the semi-closed nature of university environments, where the potential for person-to-person transmission is higher [35,36]. Although our study did not assess the carriage prevalence of MenW:cc11 among MSM individuals, our findings suggest that dense social and contact networks within the MSM community, potentially characterized by partner concurrency and relatively closed structures, could similarly facilitate the sustained circulation of this novel variant from person to person [37,38]. Previous work has demonstrated the effectiveness of vaccination strategies targeting key reservoirs of meningococcal transmission, such as adolescents, in reducing carriage, interrupting transmission and decreasing overall disease burden in all age groups through herd protection [34,39,42]. In this context, although the circulation of the ‘2020 strain’ variant has been less frequently detected in other population groups, potentially reflecting differences in screening intensity, its identification and transmission within specific MSM networks underscore the importance of monitoring the invasive potential of strains detected in non-invasive samples to determine whether similar targeted preventive strategies may eventually be warranted.

Regarding the antimicrobial susceptibility of the ‘2020 strain’ variant, our study revealed that *penA*9 was the main determinant conferring PEN^R^, as previously demonstrated [22,23]. Phylogenetic reconstruction indicated that this determinant was vertically inherited from the ‘2015 strain’ around 2011. AZ^R^ was mainly associated with modifications in the *mtrCDE* efflux operon, particularly in *mtrD*, the transcriptional repressor *mtrR* and its promoter. Most ‘2020 strain’ isolates carried a premature stop codon in *mtrR* and a −7G deletion in its promoter, consistent with the loss of MtrR function and upregulation of the MtrCDE pump. In *Ng*, loss-of-function mutations in *mtrR* increase MtrCDE expression, contributing to AZ^R^ and reduced penicillin susceptibility, supporting the mechanisms identified in this study [43,44]. Additionally, previous studies have suggested only a minor role of the MtrCDE efflux system in CIP^R^ in *Nm*, which could explain the two sporadic CIP^R^ isolates observed in our collection, both displaying a wild-type *gyrA* and MICs only one dilution above the clinical breakpoint. However, these studies also indicate that alterations in the MtrCDE system are typically found in combination with the T91I mutation in *gyrA*, acting as a secondary modulator rather than primary resistance determinants [45,46]. Therefore, the potential contribution of the MtrCDE efflux system to CIP^R^ in *Nm* requires further investigation, as most studies addressing this phenomenon have mainly focused on *Ng*.

Interestingly, five ‘2020 strain’ isolates harbouring a frameshift in *mtrC* remained susceptible to both antimicrobials, despite carrying AZ^R^-associated mutations and *penA*9. Loss of *mtrC* function has previously been linked to increased susceptibility to both azithromycin and penicillin in *Ng*, likely explaining the phenotype observed in this study [44,47]. The *mtrC* frameshift occurred in five phylogenetically distinct isolates, suggesting independent acquisition rather than vertical inheritance. The mutation occurred within a GC hexarepeat, pointing to a mutational hotspot prone to recurrent events, as previously described in pathogenic *Neisseria*, particularly in *Ng* and in urogenital-adapted lineages of *Nm* [48]. This pattern may reflect selective pressure favouring loss of MtrC, possibly as an adaptation to the urogenital tract and/or due to the fitness cost associated with constitutive MtrCDE overexpression, an effect previously documented in *Ng* and plausibly operating in *Nm* [49]. Together, these findings indicate that the MtrCDE efflux pump and its transcriptional repressor are key contributors to the resistant phenotype in this variant.

In conclusion, our study identified a novel MenW:cc11 variant, designated the ‘2020 strain’, mainly obtained from respiratory, urogenital and anorectal samples belonging to MSM individuals, and also associated with IMD cases outside this population group. Importantly, this variant exhibited resistance to penicillin, associated with the mosaic *penA*9 allele, and to azithromycin, likely driven by alterations in the MtrCDE efflux pump and its transcriptional repressor *mtrR*. Continued phenotypic and genomic surveillance of antimicrobial resistance and evolutionary dynamics in meningococcal populations, particularly among hypervirulent strains such as cc11, remains essential for early detection of emergent variants and for guiding timely public health interventions.

## Supplementary material

10.1099/mgen.0.001703 Uncited Supplementary Material 1.

## Abbreviations

cARC: mixed continuous additive relaxed clock
cc11: *Neisseria meningitidis* clonal complex 11
cc: clonal complexes
DIC: deviance information criterion
HPD: highest posterior density
IMD: invasive meningococcal disease
MICs: minimum inhibitory concentrations
MLST: multilocus sequence typing
MRCA: most recent common ancestor
MSM: men who have sex with men
*Ng*: *Neisseria gonorrhoeae*
*Nm*: *Neisseria meningitidis*
PBP2: penicillin-binding protein 2
ST: sequence type
STI: sexually transmitted infection
WGS: whole-genome sequencing
