# Supplementary material for: Genomic and evolutionary insights into a novel Neisseria meningitidis genogroup W clonal complex 11 variant with dual resistance to penicillin and azithromycin in Spain
Source: Microb Genom. 2026 May 12;12(5):001703. doi: 10.1099/mgen.0.001703 (PMC13167043; doi:10.1099/mgen.0.001703)
Supplement: Uncited Supplementary Material 1. [file mgen-12-01703-s001.pdf]

Supplementary Material

Genomic and evolutionary insights into a novel *Neisseria meningitidis* genogroup W clonal complex 11 variant with dual resistance to penicillin and azithromycin in Spain

Josep Roca-Grande (https://orcid.org/0000-0002-7177-1684)[1,2,3], Albert Moreno-Mingorance[1,3], Patricia Álvarez-López[4], Maider Arando[4], Alba Bellés-Bellés[5], Jorge Calvo-Montes[3,6], Jordi Càmara[7,8], Emilia Cercenado[8,9], Vicente Descalzo[4], M. Ángeles Galán-Ladero[10], Jorge Néstor García-Pérez[4], Frederic Gómez[11], Yannick Hoyos-Mallecot[12], Joan López-Madueño[13], Mayli Lung[12], Andrea Martín-Nalda[14], Alba Mir-Cros[3], Carmen Muñoz-Almagro[15,16,17], Daniel Navarro de la Cruz[18], Inés Oliveira-Souto[3,19], M. Ángeles Orellana[20], Begoña Palop[21], Amaresh Pérez-Argüello[15,16], Mar Olga Pérez-Moreno[22], Guillem Puigsech-Boixeda[2,3], M. Dolores Quesada[23], Alba Rivera[3,24], Carlos Rodrigo[25], Ana Rodriguez-Fernandez[6], Enrique Ruiz de Gopegui[26], Carolina Sarvisé[11], Núria Serre-Delcor[3,19], Pere Soler-Palacín[14], Aleix Soler-Garcia[15,16,27], Jesús Trejo-Zahínos[12], Belén Viñado[12], M. Nieves Larrosa[1,2,3,12,*], Juan José González-López (https://orcid.org/0000-0003-2419-5909)[1,2,3,12,*]

Affiliations

1. Microbiology Research Group, Institut de Recerca Vall d'Hebron (VHIR), Barcelona, Spain
2. Departament de Genètica i Microbiologia, Universitat Autònoma de Barcelona (UAB), Bellaterra, Spain
3. CIBER de Enfermedades Infecciosas (CIBERINFEC), Instituto de Salud Carlos III, Madrid, Spain
4. Infectious Diseases, STI/HIV Unit, Hospital Universitari Vall d'Hebron, Barcelona, Spain
5. Department of Clinical Microbiology, Hospital Universitari Arnau de Vilanova, Institut de Recerca Biomèdica de Lleida – Fundació Dr. Pifarré, IRBLleida, Av. Alcalde Rovira Roure, 80, 25198, Lleida, Spain
6. Department of Clinical Microbiology, Hospital Universitario Marqués de Valdecilla-IDIVAL, Santander, Spain
7. Department of Clinical Microbiology, Hospital Universitari de Bellvitge, IDIBELL-UB, L'Hospitalet de Llobregat, Spain
8. CIBER de Enfermedades Respiratorias (CIBERES), Instituto de Salud Carlos III, Madrid, Spain
9. Department of Clinical Microbiology and Infectious Disease, Hospital General Universitario Gregorio Marañón, Madrid, Spain
10. Department of Clinical Microbiology, Hospital Universitario Reina Sofía, Córdoba, Spain
11. Department of Clinical Microbiology, Hospital Universitari de Tarragona Joan XXIII, Pere Virgili Health Research Institute (IISPV), Tarragona, Spain
12. Department of Clinical Microbiology, Hospital Universitari Vall d'Hebron (HUVH), Barcelona, Spain
13. Fundació Althaia, Xarxa Assistencial Universitària Manresa, Manresa, Spain
14. Pediatric Infectious Diseases and Immunodeficiencies Unit, Children's Hospital, Hospital Universitari Vall d'Hebron Campus, Barcelona, Spain
15. Infectious Diseases and Microbiome Research Group, Institut de Recerca Sant Joan de Déu, Hospital Sant Joan de Déu, Esplugues, Spain
16. CIBER de Epidemiología y Salud Pública (CIBERESP), Instituto de Salud Carlos III, Madrid, Spain
17. School of Medicine and Health Sciences, Universitat Internacional de Catalunya (UIC), Barcelona, Spain
18. Department of Clinical Microbiology, Complejo Hospitalario Universitario de Santiago, Santiago de Compostela, Spain
19. Tropical Medicine Unit Vall d'Hebron-Drassanes, Infectious Diseases Department, Vall d'Hebron University Hospital, PROSICS Barcelona, Barcelona, Spain
20. Department of Clinical Microbiology, Hospital Universitario 12 de Octubre, Madrid, Spain
21. Department of Clinical Microbiology, Hospital Regional Universitario de Málaga, Málaga, Spain
22. Department of Clinical Microbiology, Hospital Universitari de Tortosa Verge de la Cinta, Tortosa, Spain
23. Department of Clinical Microbiology, Hospital Universitari Germans Trias i Pujol, UAB, Badalona, Spain
24. Department of Clinical Microbiology, Hospital de la Santa Creu i Sant Pau, Sant Pau Biomedical Research Institute (IIB Sant Pau), Barcelona, Spain
25. Department of Paediatrics, Hospital Universitari Germans Trias i Pujol, Universitat Autònoma de Barcelona (UAB), Badalona, Spain.
26. Department of Clinical Microbiology, Hospital Universitario Son Espases, Instituto de Investigación Sanitaria Illes Balears (IdISBa), Palma de Mallorca, Spain
27. Department of Pediatrics, Hospital Sant Joan de Déu, Esplugues, Spain

* These authors contributed equally to the work and share last authorship

Correspondence: Juan José González-López (juanjo.gonzalez@vallhebron.cat)

Contents:

Supplementary Figure 1	Page 2
Supplementary Figure 2	Page 3
Supplementary Figure 3	Page 4
Supplementary Table 1	Page 5
Supplementary Table 2	Page 10
Supplementary Table 3	Page 11

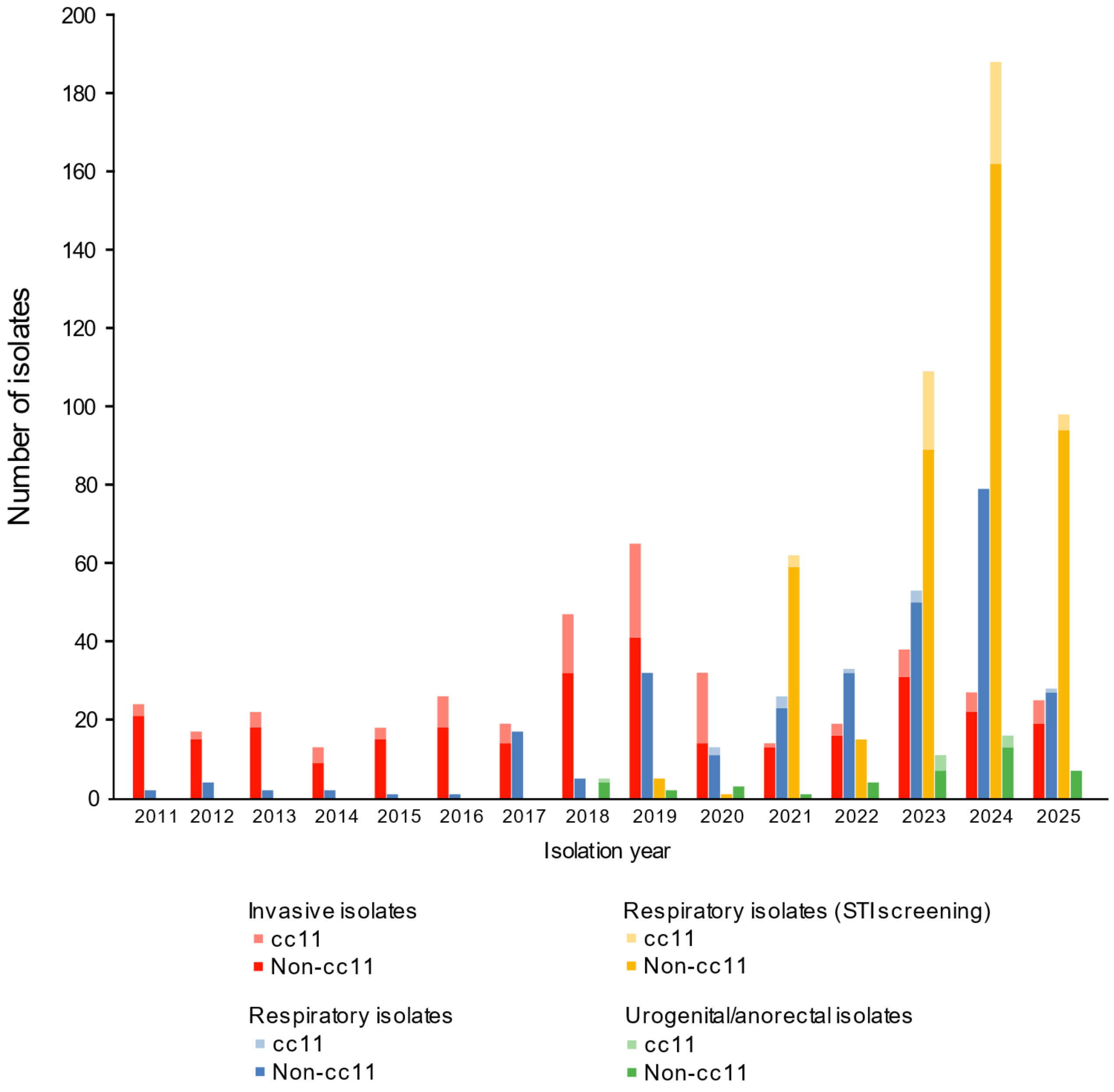

Supplementary Figure 1. Temporal distribution of the full *Neisseria meningitidis* collection from this study comprising 1,226 isolates, classified by year of isolation, sampling source, and cc11 status.

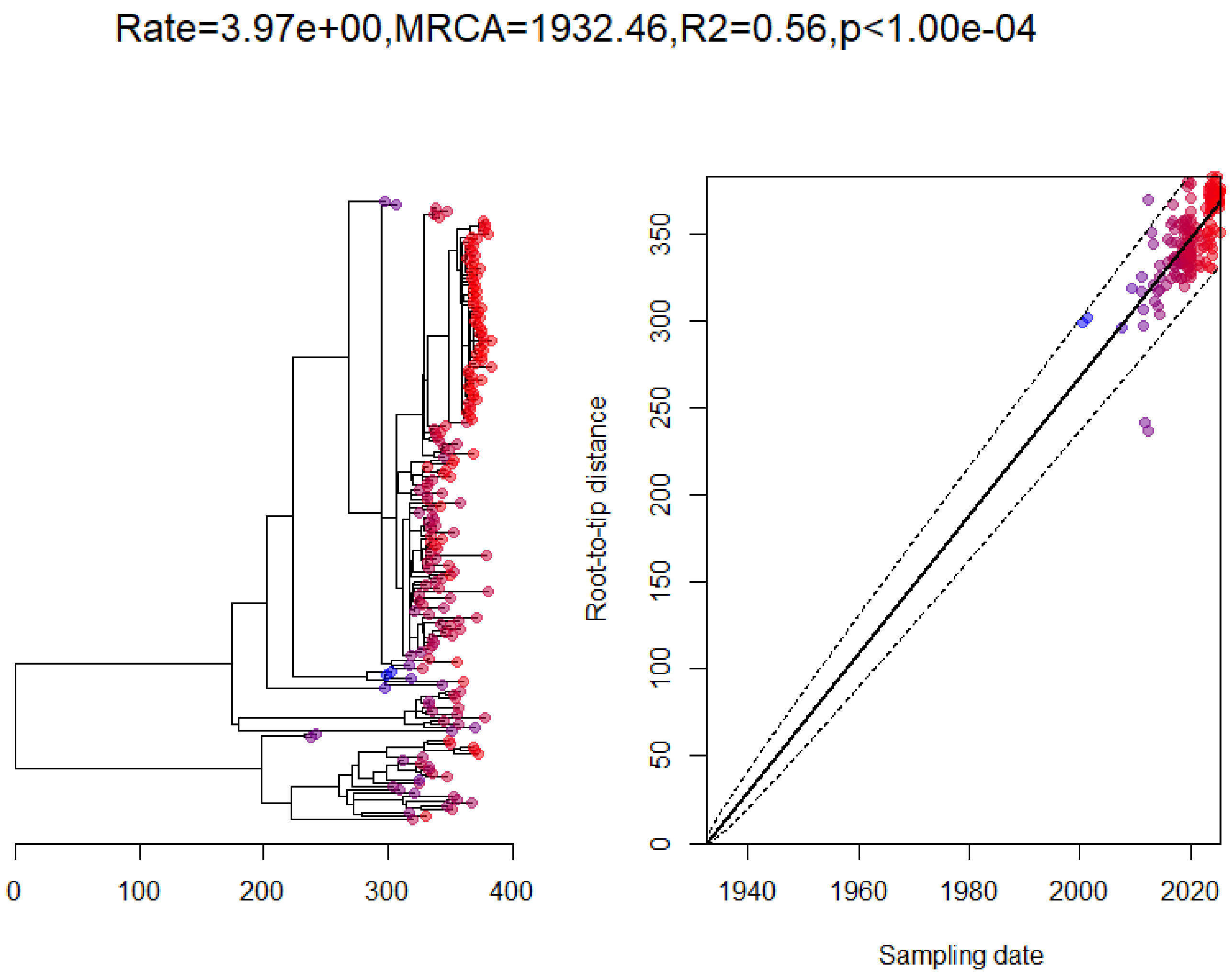

Supplementary Figure 2. Root-to-tip regression analysis showing temporal signal in the 179 *Neisseria meningitidis* cc11 dataset.

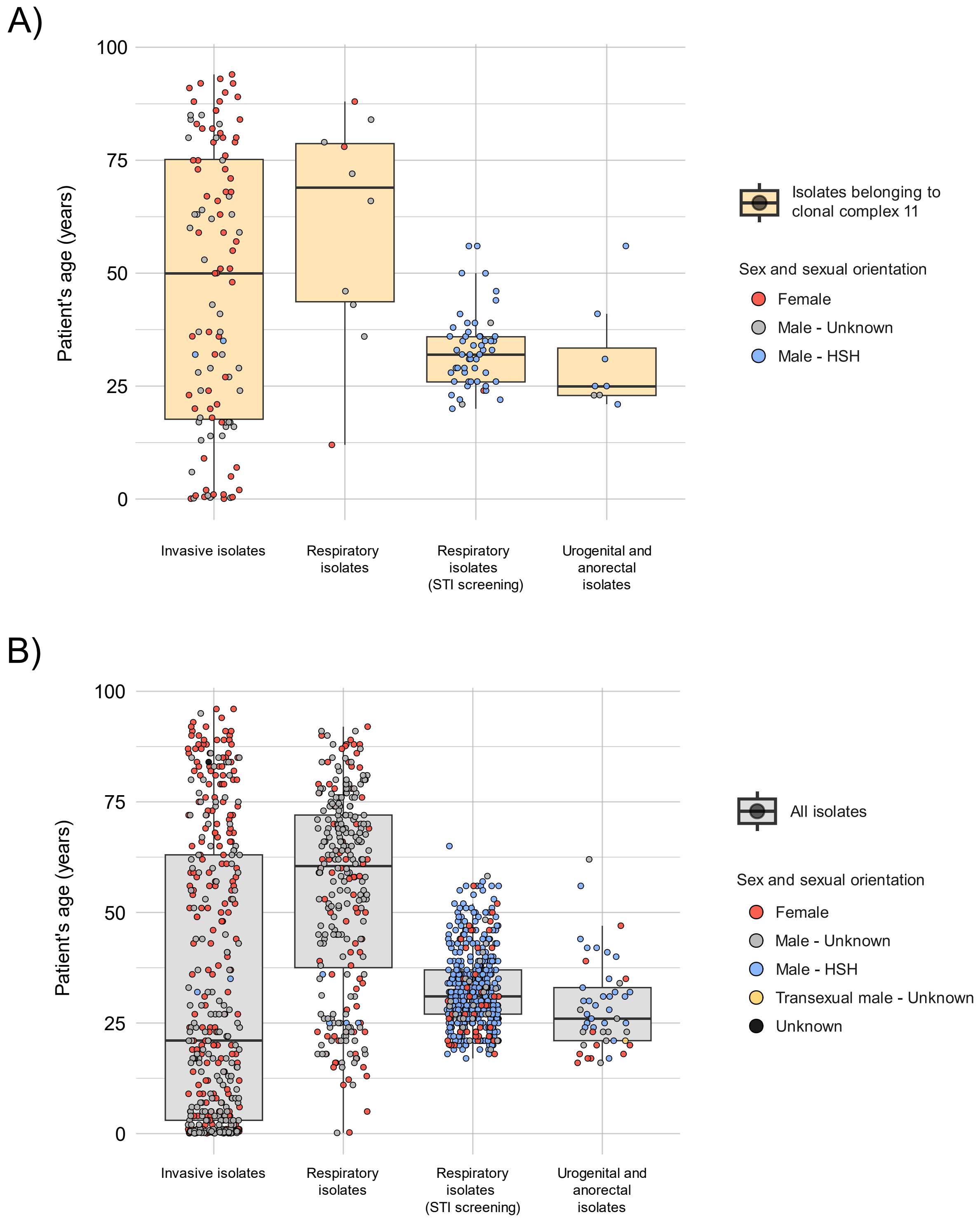

Supplementary Figure 3. Age distribution of individuals from whom *Neisseria meningitidis* isolates were recovered, according to sampling source: (A) clonal complex 11 isolates and (B) the full collection of 1,226 isolates. Boxplots represent the median (central line), interquartile range (boxes), and 1.5× interquartile range (whiskers). Individual data points correspond to single individuals and are shown as circles, colour-coded by sex and sexual orientation.

Supplementary Table 1. List of *Neisseria meningitidis* genomes belonging to clonal complex 11 identified and analyzed in this study, including NCBI and PubMLST identifiers, as well as additional data.

PubMLST ID	Lineage	Sublineage	Genetic variant	Isolate	Run Accession	Biosample Accession	Isolation year	Age, sex and sexual orientation[1]	Sample type and specimen	STI screening	Genogroup, ST and CC	Penicillin susceptibility	*penA* allele and mutations	Azithromycin susceptibility	*mtrC* allele and mutations	*mtrD* allele and mutations	*mtrE* allele	*mtrR* allele and mutations	*promoter_mtrR allele and mutations*
63357	11.1	South American/UK	2015 strain	Nmen108	SRR36379784	SAMN53825784	2019	80, Female, NA	Invasive (Blood)	No	W:ST11:CC11	R	9 (F504L, A510V, I515V, H541N, I566V)	S	2 (WT)	77 (F854L)	1	2 (WT)	5 (WT)
63358	11.2	NA	NA	Nmen109	SRR36379783	SAMN53825785	2019	37, Male, Unknown	Invasive (Blood)	No	C:ST11760:CC11	R	327 (F504L, A510V, I515V, I566V)	R	718 (WT)	961 (K823E, F854L)	670	318 (WT)	5 (WT)
63359	11.1	South American/UK	2013 strain	Nmen118	SRR36379782	SAMN53825786	2019	75, Male, Unknown	Invasive (Blood)	No	W:ST11:CC11	S	1 (WT)	S	1 (WT)	1 (WT)	1	989 (WT)	5 (WT)
63360	11.1	South American/UK	2015 strain	Nmen120	SRR36379780	SAMN53825787	2019	68, Female, NA	Invasive (Blood)	No	W:ST11:CC11	R	9 (F504L, A510V, I515V, H541N, I566V)	S	2 (WT)	77 (F854L)	1	2 (WT)	5 (WT)
63362	11.1	South American/UK	2015 strain	Nmen123	SRR36379779	SAMN53825788	2019	29, Male, Unknown	Invasive (Blood)	No	W:ST11:CC11	R	9 (F504L, A510V, I515V, H541N, I566V)	S	2 (WT)	77 (F854L)	1	2 (WT)	5 (WT)
63363	11.1	South American/UK	UK strain	Nmen124	SRR36379778	SAMN53825789	2019	1m, Female, NA	Invasive (Blood)	No	W:ST14955:CC11	S	1 (WT)	S	1 (WT)	1 (WT)	1	1 (WT)	5 (WT)
70761	11.1	South American/UK	2013 strain	Nmen121	SRR36394163	SAMN53856171	2019	73, Female, NA	Invasive (Blood)	No	W:ST11:CC11	S	1 (WT)	S	1 (WT)	1 (WT)	1	989 (WT)	5 (WT)
71925	11.1	South American/UK	2013 strain	Nmen132	SRR36394162	SAMN53856172	2019	20, Female, NA	Invasive (Blood)	No	W:ST11:CC11	S	1 (WT)	S	1 (WT)	1 (WT)	1	989 (WT)	5 (WT)
72249	11.1	South American/UK	2013 strain	Nmen139	SRR36394161	SAMN53856173	2020	86, Female, NA	Invasive (Blood)	No	W:ST11:CC11	S	1 (WT)	S	1 (WT)	1 (WT)	1	989 (WT)	5 (WT)
72250	11.1	South American/UK	2013 strain	Nmen141	SRR36394160	SAMN53856174	2020	62, Male, Unknown	Invasive (Blood)	No	W:ST11:CC11	S	1 (WT)	S	1 (WT)	1 (WT)	1	989 (WT)	5 (WT)
72254	11.1	NA	Proximal region	Nmen148	SRR36394159	SAMN53856175	2019	84, Female, NA	Invasive (Blood)	No	C:ST11:CC11	S	1 (WT)	S	1 (WT)	1 (WT)	1	1 (WT)	5 (WT)
72758	11.1	South American/UK	2013 strain	Nmen146	SRR36379777	SAMN53825790	2020	16, Male, Unknown	Invasive (Blood)	No	W:ST11:CC11	S	1 (WT)	S	1 (WT)	1 (WT)	1	989 (WT)	5 (WT)
72759	11.1	South American/UK	2013 strain	Nmen150	SRR36379776	SAMN53825791	2020	57, Female, NA	Invasive (Blood)	No	W:ST11:CC11	S	1 (WT)	S	1 (WT)	1 (WT)	1	989 (WT)	5 (WT)
72761	11.1	South American/UK	2020 strain	Nmen152	SRR36379775	SAMN53825792	2020	29, Male, Unknown	Invasive (Blood)	No	W:ST11:CC11	R	9 (F504L, A510V, I515V, H541N, I566V)	R	493 (WT)	4108 (K823E, F854L)	1	3151 (mtrR_1:c.337delT (p.Leu114*))	16 (-7G deletion)
72889	11.1	South American/UK	2013 strain	Nmen157	SRR36379774	SAMN53825793	2020	50, Female, NA	Invasive (Blood)	No	W:ST11:CC11	S	1 (WT)	S	1 (WT)	1 (WT)	1	989 (WT)	5 (WT)
72891	11.2	NA	NA	Nmen161	SRR36379772	SAMN53825795	2020	51, Female, NA	Invasive (Blood)	No	C:ST11:CC11	R	248 (F504L, A510V, I515V, H541N, I566V)	S	147 (WT)	2927 (WT)	2	1 (WT)	5 (WT)
72892	11.1	South American/UK	2013 strain	Nmen162	SRR36379771	SAMN53825796	2020	59, Female, NA	Invasive (Blood)	No	W:ST11:CC11	S	1 (WT)	S	1 (WT)	1 (WT)	1	989 (WT)	5 (WT)
77201	11.1	South American/UK	2013 strain	Nmen164	SRR36394157	SAMN53856177	2020	2m, Male, Unknown	Invasive (Blood)	No	W:ST11:CC11	S	1 (WT)	S	1 (WT)	1 (WT)	1	989 (WT)	5 (WT)
77202	11.2	NA	NA	Nmen165	SRR36394156	SAMN53856178	2020	24, Male, Unknown	Invasive (Blood)	No	C:ST11:CC11	R	248 (F504L, A510V, I515V, H541N, I566V)	S	147 (WT)	2927 (WT)	2	1 (WT)	5 (WT)
77203	11.1	South American/UK	2015 strain	Nmen166	SRR36394155	SAMN53856179	2020	88, Female, NA	Invasive (Blood)	No	W:ST11:CC11	R	9 (F504L, A510V, I515V, H541N, I566V)	R	1544 (WT)	77 (F854L)	1	3148 (mtrR_1:c.150delT (p.Leu114*))	16 (-7G deletion)
77206	11.1	South American/UK	2015 strain	Nmen173	SRR36394152	SAMN53856181	2020	37, Female, NA	Invasive (Blood)	No	W:ST11:CC11	R	9 (F504L, A510V, I515V, H541N, I566V)	S	2 (WT)	77 (F854L)	1	2 (WT)	5 (WT)
82433	11.1	NA	Proximal region	Nmen178	SRR36394151	SAMN53856182	2016	37, Male, Unknown	Invasive (Blood)	No	C:ST11:CC11	R	1 (WT)	R	1 (WT)	1 (WT)	1	1 (WT)	5 (WT)
82435	11.2	NA	NA	Nmen180	SRR36394150	SAMN53856183	2016	2, Female, NA	Invasive (Blood)	No	B:ST11:CC11	S	2 (WT)	R	1557 (WT)	150 (F854L)	70	1 (WT)	5 (WT)
82436	11.1	NA	Proximal region	Nmen181	SRR36394149	SAMN53856184	2014	17, Male, Unknown	Invasive (Blood)	No	C:ST11:CC11	S	1 (WT)	S	1 (WT)	1 (WT)	1	1 (WT)	5 (WT)
82437	11.1	South American/UK	2013 strain	Nmen182	SRR36394148	SAMN53856185	2015	17, Male, Unknown	Invasive (Blood)	No	W:ST11:CC11	S	1 (WT)	S	1 (WT)	1 (WT)	1	989 (WT)	5 (WT)
82439	11.2	NA	NA	Nmen184	SRR36394147	SAMN53856186	2014	36, Female, NA	Invasive (Blood)	No	C:ST11:CC11	S	7 (F504L, A510V, I515V, H541N, I566V)	S	2 (WT)	2 (WT)	2	2 (WT)	5 (WT)
82444	11.1	South American/UK	2013 strain	Nmen189	SRR36394146	SAMN53856187	2016	86, Female, NA	Invasive (Blood)	No	W:ST11:CC11	S	1 (WT)	S	1 (WT)	1 (WT)	1	989 (WT)	5 (WT)
82449	11.1	South American/UK	UK strain	Nmen194	SRR36394145	SAMN53856188	2017	92, Female, NA	Invasive (Blood)	No	W:ST10651:CC11	S	1 (WT)	S	1 (WT)	1 (WT)	1	1 (WT)	5 (WT)
82452	11.1	South American/UK	2013 strain	Nmen197	SRR36394144	SAMN53856189	2018	83, Female, NA	Invasive (CSF)	No	W:ST11:CC11	S	1 (WT)	S	1 (WT)	1 (WT)	1	989 (WT)	5 (WT)
82459	11.1	South American/UK	2013 strain	Nmen204	SRR36394143	SAMN53856190	2016	14, Male, Unknown	Invasive (CSF)	No	W:ST11:CC11	S	1 (WT)	S	1 (WT)	1 (WT)	1	989 (WT)	5 (WT)
82465	11.1	South American/UK	2013 strain	Nmen211	SRR36394141	SAMN53856191	2018	81, Female, NA	Invasive (Blood)	No	W:ST11:CC11	S	1 (WT)	S	1 (WT)	1 (WT)	1	989 (WT)	5 (WT)
82466	11.1	South American/UK	2013 strain	Nmen212	SRR36394140	SAMN53856192	2017	17, Female, NA	Invasive (Blood)	No	W:ST11:CC11	S	1 (WT)	S	1 (WT)	1 (WT)	1	989 (WT)	5 (WT)
82469	11.1	South American/UK	2013 strain	Nmen215	SRR36394139	SAMN53856193	2018	93, Female, NA	Invasive (Blood)	No	W:ST11:CC11	S	1 (WT)	S	1 (WT)	1 (WT)	1	989 (WT)	5 (WT)
82474	11.1	South American/UK	2013 strain	Nmen220	SRR36394138	SAMN53856194	2018	1, Female, NA	Invasive (Blood)	No	W:ST11:CC11	S	1 (WT)	S	1 (WT)	1 (WT)	1	989 (WT)	5 (WT)
82478	11.2	NA	NA	Nmen224	SRR36394137	SAMN53856195	2018	60, Male, Unknown	Invasive (Blood)	No	C:ST11:CC11	S	2 (WT)	S	147 (WT)	257 (WT)	2	1 (WT)	5 (WT)
82480	11.2	NA	NA	Nmen226	SRR36394136	SAMN53856196	2017	9, Female, NA	Invasive (Blood)	No	C:ST11:CC11	S	3 (WT)	S	2 (WT)	2 (WT)	159	2 (WT)	5 (WT)
82481	11.1	South American/UK	UK strain	Nmen227	SRR36394135	SAMN53856197	2017	75, Female, NA	Invasive (Blood)	No	W:ST10651:CC11	S	1 (WT)	S	1 (WT)	1 (WT)	1	1 (WT)	5 (WT)
82482	11.1	South American/UK	2013 strain	Nmen228	SRR36394134	SAMN53856198	2018	18, Male, Unknown	Invasive (CSF)	No	W:ST14822:CC11	S	1 (WT)	S	1 (WT)	1 (WT)	1	989 (WT)	5 (WT)
82483	11.1	African/Hajj outbreak	NA	Nmen229	SRR36394133	SAMN53856199	2013	9m, Male, Unknown	Invasive (Blood)	No	W:ST11:CC11	S	1 (WT)	S	1 (WT)	1 (WT)	1	1 (WT)	5 (WT)
82486	11.2	NA	NA	Nmen232	SRR36394132	SAMN53856200	2013	32, Male, Unknown	Invasive (CSF)	No	C:ST11:CC11	S	248 (F504L, A510V, I515V, H541N, I566V)	S	147 (WT)	257 (WT)	2	1 (WT)	5 (WT)

82489	11.2	NA	NA	Nmen235	SRR36394240	SAMN53856201	2014	32, Female, NA	Invasive (CSF)	No	C:ST11:CC11	S	7 (F504L, A510V, I515V, H541N, I566V)	S	1593 (WT)	65 (WT)	3	2 (WT)	5 (WT)
82493	11.1	South American/UK	2013 strain	Nmen239	SRR36394239	SAMN53856202	2018	89, Female, NA	Invasive (Blood)	No	W:ST11:CC11	S	1 (WT)	S	1 (WT)	1 (WT)	1	989 (WT)	5 (WT)
82498	11.1	South American/UK	South American	Nmen244	SRR36394238	SAMN53856203	2011	1m, Female, NA	Invasive (Blood)	No	W:ST11:CC11	S	1 (WT)	S	1 (WT)	1 (WT)	1	1 (WT)	5 (WT)
82515	11.2	NA	NA	Nmen262	SRR36394237	SAMN53856204	2013	16, Male, Unknown	Invasive (CSF)	No	C:ST10140:CC11	R	7 (F504L, A510V, I515V, H541N, I566V)	S	2 (WT)	2 (WT)	2	2 (WT)	5 (WT)
82519	11.1	South American/UK	2015 strain	Nmen267	SRR36394236	SAMN53856205	2020	20, Female, NA	Invasive (Blood)	No	W:ST11:CC11	R	9 (F504L, A510V, I515V, H541N, I566V)	S	2 (WT)	77 (F854L)	1	2 (WT)	5 (WT)
82520	11.1	South American/UK	2013 strain	Nmen268	SRR36394235	SAMN53856206	2020	79, Female, NA	Invasive (Blood)	No	W:ST11:CC11	S	1 (WT)	S	1 (WT)	1 (WT)	1	989 (WT)	5 (WT)
82526	11.1	South American/UK	2013 strain	Nmen274	SRR36394234	SAMN53856207	2018	1, Female, NA	Invasive (CSF)	No	W:ST11:CC11	S	1 (WT)	S	1 (WT)	1 (WT)	1	989 (WT)	5 (WT)
82530	11.2	NA	NA	Nmen278	SRR36394233	SAMN53856208	2015	63, Male, Unknown	Invasive (CSF)	No	C:ST11:CC11	R	327 (F504L, A510V, I515V, I566V)	S	2 (WT)	2 (WT)	159	2 (WT)	5 (WT)
82538	11.2	NA	NA	Nmen286	SRR36394232	SAMN53856209	2014	7, Female, NA	Invasive (Blood)	No	C:ST1237:CC11	S	2 (WT)	R	147 (WT)	257 (WT)	2	133 (WT)	5 (WT)
85382	11.2	NS	NA	Nmen12	SRR36394165	SAMN53856170	2019	36, Female, NA	Invasive (Blood)	No	C:ST11:CC11	R	248 (F504L, A510V, I515V, H541N, I566V)	S	147 (WT)	257 (WT)	2	1 (WT)	5 (WT)
85385	11.1	South American/UK	2013 strain	Nmen47	SRR36379765	SAMN53825801	2019	90, Female, NA	Invasive (Blood)	No	W:ST11:CC11	S	1 (WT)	S	1 (WT)	1 (WT)	1	989 (WT)	5 (WT)
85389	11.1	South American/UK	2013 strain	Nmen22	SRR36379769	SAMN53825797	2018	23, Male, Unknown	Urogenital or anorectal (Urethral swab)	Yes	NG:ST11:CC11	S	1 (WT)	S	89 (WT)	141 (F854L)	2112	27 (WT)	5 (WT)
88871	11.1	South American/UK	2013 strain	Nmen3	SRR36379768	SAMN53825798	2019	59, Male, Unknown	Invasive (Blood)	No	W:ST11:CC11	S	1 (WT)	S	1 (WT)	1 (WT)	1	989 (WT)	5 (WT)
88872	11.1	South American/UK	2013 strain	Nmen6	SRR36379760	SAMN53825806	2018	6m, Female, NA	Invasive (Blood)	No	W:ST11:CC11	S	1 (WT)	S	1 (WT)	1 (WT)	1	989 (WT)	5 (WT)
88878	11.1	South American/UK	2013 strain	Nmen55	SRR36379763	SAMN53825803	2019	63, Female, NA	Invasive (Blood)	No	W:ST11:CC11	S	1 (WT)	S	1 (WT)	1 (WT)	1	989 (WT)	5 (WT)
88879	11.1	South American/UK	2013 strain	Nmen56	SRR36379762	SAMN53825804	2019	82, Female, NA	Invasive (Blood)	No	W:ST11:CC11	S	1 (WT)	S	1 (WT)	1 (WT)	1	1 (WT)	5 (WT)
88881	11.1	South American/UK	2013 strain	Nmen58	SRR36379761	SAMN53825805	2018	17, Male, Unknown	Invasive (Blood)	No	W:ST11:CC11	S	1 (WT)	S	1 (WT)	1 (WT)	1	989 (WT)	5 (WT)
89658	11.1	South American/UK	2013 strain	Nmen63	SRR36379758	SAMN53825807	2019	9m, Female, NA	Invasive (Blood)	No	W:ST11:CC11	S	1 (WT)	S	1 (WT)	1 (WT)	1	989 (WT)	5 (WT)
89661	11.1	South American/UK	2013 strain	Nmen67	SRR36379752	SAMN53825808	2019	80, Female, NA	Invasive (Blood)	No	W:ST11:CC11	S	1 (WT)	S	1 (WT)	1 (WT)	1	989 (WT)	5 (WT)
89664	11.1	NA	Proximal region	Nmen48	SRR36379764	SAMN53825802	2019	85, Male, Unknown	Invasive (CSF)	No	C:ST14956:CC11	S	1 (WT)	S	1110 (WT)	1 (WT)	1	1 (WT)	5 (WT)
91800	11.1	South American/UK	2015 strain	Nmen89	SRR36379753	SAMN53825809	2019	88, Female, NA	Invasive (Blood)	No	W:ST11:CC11	R	9 (F504L, A510V, I515V, H541N, I566V)	S	2 (WT)	77 (F854L)	1	2 (WT)	5 (WT)
93847	11.1	South American/UK	2015 strain	Nmen101	SRR36394166	SAMN53856169	2019	17, Male, Unknown	Invasive (Blood and CSF)	No	W:ST11:CC11	R	9 (F504L, A510V, I515V, H541N, I566V)	S	2 (WT)	77 (F854L)	1	2 (WT)	5 (WT)
102634	11.1	South American/UK	2013 strain	Nmen160	SRR36379773	SAMN53825794	2020	2, Female, NA	Invasive (Joint fluid)	No	W:ST11:CC11	S	1 (WT)	S	1685 (WT)	1 (WT)	1	989 (WT)	5 (WT)
110125	11.2	NA	NA	DE026	SRR36379754	SAMN53825810	2021	33, Male, MSM	Respiratory (Throat swab)	Yes	C:ST11:CC11	R	7 (F504L, A510V, I515V, H541N, I566V)	S	147 (WT)	257 (WT)	2	310 (WT)	5 (WT)
110156	11.1	South American/UK	South American	DE070	SRR36379755	SAMN53825811	2021	23, Male, MSM	Respiratory (Throat swab)	Yes	W:ST11:CC11	R	952 (F504L, A510V, I515V, H541N, I566V)	S	1 (WT)	1 (WT)	1	1 (WT)	5 (WT)
115307	11.1	South American/UK	UK strain	Nmen344	SRR36379767	SAMN53825799	2021	92, Female, NA	Invasive (Blood)	No	W:ST10651:CC11	S	1 (WT)	R	1840 (WT)	5799 (K823E)	1	1559 (D79N)	22 (-7G deletion)
115309	11.1	South American/UK	2015 strain	Nmen346	SRR36379766	SAMN53825800	2022	94, Female, NA	Invasive (Blood)	No	W:ST11:CC11	R	9 (F504L, A510V, I515V, H541N, I566V)	S	2 (WT)	77 (F854L)	1	2 (WT)	5 (WT)
122674	11.1	South American/UK	2013 strain	H08-EMI-001	SRR36394205	SAMN53856133	2017	48, Female, NA	Invasive (CSF)	No	W:ST11:CC11	S	1 (WT)	S	1 (WT)	1 (WT)	1	989 (WT)	5 (WT)
123082	11.1	South American/UK	2013 strain	H15-EMI-005	SRR36394179	SAMN53856157	2022	28, Male, Unknown	Invasive (Blood)	No	W:ST11:CC11	S	1 (WT)	S	1 (WT)	1 (WT)	1	989 (WT)	5 (WT)
123232	11.1	South American/UK	2013 strain	H03-EMI-001	SRR36394210	SAMN53856129	2018	76, Female, NA	Invasive (Blood)	No	W:ST11:CC11	S	1 (WT)	S	1 (WT)	1 (WT)	1	989 (WT)	5 (WT)
123233	11.1	South American/UK	2013 strain	H08-EMI-005	SRR36394204	SAMN53856134	2018	84, Male, Unknown	Invasive (Blood)	No	W:ST11:CC11	S	90 (F504L, A510V, I515V, H541N, I566V)	S	1 (WT)	1 (WT)	1	989 (WT)	5 (WT)
123236	11.1	NA	Proximal region	H08-EMI-011	SRR36394203	SAMN53856135	2019	24, Female, NA	Invasive (Blood)	No	C:ST11:CC11	S	1 (WT)	S	1 (WT)	1 (WT)	1	1 (WT)	5 (WT)
123238	11.1	South American/UK	UK strain	H08-EMI-016	SRR36394202	SAMN53856136	2020	21, Female, NA	Invasive (Blood)	No	W:ST11:CC11	R	1 (WT)	S	1 (WT)	1 (WT)	1	1 (WT)	5 (WT)
123241	11.2	NA	NA	H09-EMI-012	SRR36394201	SAMN53856137	2012	71, Female, NA	Invasive (CSF)	No	C:ST11:CC11	S	3 (WT)	S	2 (WT)	4273 (WT)	2	2 (WT)	5 (WT)
123242	11.2	NA	NA	H09-EMI-014	SRR36394200	SAMN53856138	2011	41, Male, Unknown	Invasive (CSF)	No	C:ST11:CC11	S	3 (WT)	S	2 (WT)	4273 (WT)	2	2 (WT)	5 (WT)
123246	11.1	South American/UK	2013 strain	H12-EMI-001	SRR36394190	SAMN53856147	2020	63, Male, Unknown	Invasive (Blood)	No	W:ST11:CC11	S	1 (WT)	S	1 (WT)	1 (WT)	1	989 (WT)	5 (WT)
123247	11.1	South American/UK	UK strain	H12-EMI-002	SRR36394189	SAMN53856148	2019	4m, Male, Unknown	Invasive (CSF)	No	W:ST11:CC11	S	1 (WT)	S	1 (WT)	1 (WT)	1	1 (WT)	5 (WT)
123248	11.1	South American/UK	2013 strain	H12-EMI-003	SRR36394188	SAMN53856149	2019	27, Male, Unknown	Invasive (Blood)	No	W:ST11:CC11	S	1 (WT)	S	1 (WT)	1 (WT)	1	989 (WT)	5 (WT)
123249	11.1	South American/UK	2013 strain	H12-EMI-005	SRR36394187	SAMN53856150	2019	14, Male, Unknown	Invasive (Blood)	No	W:ST11:CC11	S	1 (WT)	S	1 (WT)	1 (WT)	1	989 (WT)	5 (WT)
123251	11.2	NA	NA	H14-EMI-002	SRR36394185	SAMN53856151	2011	13, Male, Unknown	Invasive (CSF)	No	C:ST11:CC11	R	248 (F504L, A510V, I515V, H541N, I566V)	S	147 (WT)	257 (WT)	2	1 (WT)	5 (WT)
123252	11.1	NA	Proximal region	H14-EMI-008	SRR36394184	SAMN53856152	2016	27, Female, NA	Invasive (Blood)	No	C:ST11:CC11	S	1 (WT)	S	1 (WT)	1 (WT)	1	1 (WT)	5 (WT)
123253	11.1	NA	Proximal region	H14-EMI-009	SRR36394183	SAMN53856153	2018	4m, Male, Unknown	Invasive (CSF)	No	C:ST11:CC11	S	1 (WT)	S	1110 (WT)	1 (WT)	1	1 (WT)	5 (WT)
123254	11.1	South American/UK	UK strain	H14-EMI-010	SRR36394182	SAMN53856154	2019	6, Male, Unknown	Invasive (Blood)	No	W:ST11:CC11	S	1 (WT)	S	1 (WT)	1 (WT)	1	1 (WT)	5 (WT)

123255	11.1	NA	Proximal region	H14-EMI-012	SRR36394181	SAMN53856155	2016	43, Male, Unknown	Invasive (Blood)	No	C:ST11:CC11	S	1 (WT)	S	1 (WT)	1 (WT)	1	1 (WT)	5 (WT)
127374	11.1	South American/UK	2015 strain	H15-EMI-016	SRR36394178	SAMN53856158	2022	59, Female, NA	Invasive (Blood)	No	W:ST11:CC11	R	9 (F504L, A510V, I515V, H541N, I566V)	S	2 (WT)	77 (F854L)	1	2 (WT)	5 (WT)
127443	11.1	South American/UK	South American	Nmen168	SRR36394154	SAMN53856180	2020	84, Male, Unknown	Respiratory (Sputum)	No	W:ST11:CC11	R	9 (F504L, A510V, I515V, H541N, I566V)	S	1 (WT)	1 (WT)	1	1 (WT)	5 (WT)
127451	11.1	NA	Proximal region	Nmen306	SRR36394231	SAMN53856210	2021	72, Male, Unknown	Respiratory (Selective bronchoaspiration)	No	C:ST11:CC11	S	1 (WT)	S	1110 (WT)	1 (WT)	1	1 (WT)	5 (WT)
127464	11.1	South American/UK	2013 strain	Nmen331	SRR36394228	SAMN53856212	2021	78, Female, NA	Respiratory (Sputum)	No	NG:ST11:CC11	S	1 (WT)	S	1 (WT)	1 (WT)	1	989 (WT)	5 (WT)
128266	11.1	South American/UK	2013 strain	H15-EMI-021	SRR36394177	SAMN53856159	2023	5m, Female, NA	Invasive (Blood)	No	W:ST17527:CC11	S	1 (WT)	S	1 (WT)	1 (WT)	1	989 (WT)	5 (WT)
128268	11.1	South American/UK	2020 strain	EPD011	SRR36379756	SAMN53825812	2022	35, Male, Unknown	Respiratory (Throat swab)	No	W:ST11:CC11	R	9 (F504L, A510V, I515V, H541N, I566V)	R	493 (WT)	4108 (K823E, F854L)	1	3151 (mtrR_1:c.337delT (p.Leu114*))	16 (-7G deletion)
128275	11.1	South American/UK	2015 strain	Nmen158	SRR36394158	SAMN53856176	2020	79, Male, Unknown	Respiratory (Sputum)	No	NG:ST11:CC11	R	9 (F504L, A510V, I515V, H541N, I566V)	S	2 (WT)	77 (F854L)	1	2 (WT)	5 (WT)
128279	11.1	South American/UK	2013 strain	Nmen325	SRR36394229	SAMN53856211	2021	43, Male, Unknown	Respiratory (Sputum)	No	NG:ST11:CC11	S	1 (WT)	S	1 (WT)	1 (WT)	1	989 (WT)	5 (WT)
128292	11.1	South American/UK	2020 strain	H07-RESP-016	SRR36394207	SAMN53856131	2021	29, Male, MSM	Respiratory (Throat swab)	Yes	W:ST11:CC11	R	9 (F504L, A510V, I515V, H541N, I566V)	R	2030 (WT)	4108 (K823E, F854L)	1	1569 (D79N)	11 (-7G deletion)
128311	11.1	NA	Proximal region	H10-EMI-004	SRR36394199	SAMN53856139	2012	24, Male, Unknown	Invasive (Blood)	No	C:ST11:CC11	S	1 (WT)	S	1 (WT)	1 (WT)	1	1 (WT)	5 (WT)
128313	11.1	NA	Proximal region	H10-EMI-006	SRR36394198	SAMN53856140	2013	82, Female, NA	Invasive (Blood)	No	C:ST15701:CC11	R	9 (F504L, A510V, I515V, H541N, I566V)	S	1 (WT)	1 (WT)	1	1 (WT)	5 (WT)
128315	11.1	NA	Proximal region	H10-EMI-012	SRR36394196	SAMN53856141	2018	73, Female, NA	Invasive (Blood)	No	C:ST11:CC11	S	1 (WT)	S	1 (WT)	1 (WT)	1	1 (WT)	5 (WT)
128316	11.1	South American/UK	2013 strain	H10-EMI-013	SRR36394195	SAMN53856142	2018	55, Female, NA	Invasive (Blood)	No	W:ST11:CC11	R	1 (WT)	S	1 (WT)	1 (WT)	1	989 (WT)	5 (WT)
134818	11.1	South American/UK	2020 strain	H01-GEN-001	SRR36379739	SAMN53825814	2023	31, Male, MSM	Urogenital or anorectal (Rectal swab)	Yes	W:ST17612:CC11	R	9 (F504L, A510V, I515V, H541N, I566V)	R	493 (WT)	4108 (K823E, F854L)	1	3151 (mtrR_1:c.337delT (p.Leu114*))	16 (-7G deletion)
134831	11.2	NA	NA	H15-EMI-031	SRR36394173	SAMN53856162	2020	5, Female, NA	Invasive (Unknown)	No	B:ST11:CC11	R	248 (F504L, A510V, I515V, H541N, I566V)	S	147 (WT)	257 (WT)	2	1 (WT)	5 (WT)
134840	11.1	South American/UK	UK strain	H15-EMI-040	SRR36394172	SAMN53856163	2023	67, Female, NA	Invasive (Blood)	No	W:ST11:CC11	S	1 (WT)	S	1 (WT)	1 (WT)	1	1 (WT)	5 (WT)
134938	11.1	African/Hajj outbreak	NA	H15-EMI-024	SRR36394176	SAMN53856160	2023	66, Female, NA	Invasive (Blood)	No	W:ST11:CC11	S	1 (WT)	S	1 (WT)	1 (WT)	1	1 (WT)	5 (WT)
134940	11.1	South American/UK	UK strain	H15-EMI-026	SRR36394174	SAMN53856161	2023	79, Female, NA	Invasive (Blood)	No	W:ST17488:CC11	S	1 (WT)	S	1 (WT)	1 (WT)	1	1 (WT)	5 (WT)
135375	11.2	NA	NA	H02-EMI-002	SRR36394213	SAMN53856126	2016	35, Male, MSM	Invasive (Blood)	No	C:ST11:CC11	R	327 (F504L, A510V, I515V, I566V)	S	9 (WT)	3880 (WT)	159	2786 (WT)	7 (WT)
135379	11.2	NA	NA	H02-EMI-006	SRR36394212	SAMN53856127	2015	68, Female, NA	Invasive (Blood)	No	C:ST11:CC11	R	248 (F504L, A510V, I515V, H541N, I566V)	S	147 (WT)	257 (WT)	2	1 (WT)	5 (WT)
135380	11.2	NA	NA	H02-EMI-007	SRR36394211	SAMN53856128	2016	32, Male, MSM	Invasive (Blood and CSF)	No	C:ST11:CC11	S	327 (F504L, A510V, I515V, I566V)	S	9 (WT)	3880 (WT)	159	2042 (WT)	7 (WT)
135578	11.1	South American/UK	2020 strain	H01-RESP-006	SRR36379742	SAMN53825817	2023	32, Male, MSM	Respiratory (Throat swab)	Yes	W:ST11:CC11	R	9 (F504L, A510V, I515V, H541N, I566V)	R	493 (WT)	4108 (K823E, F854L)	1	3151 (mtrR_1:c.337delT (p.Leu114*))	16 (-7G deletion)
140807	11.2	NA	NA	H01-GEN-003	SRR36379740	SAMN53825815	2023	41, Male, MSM	Urogenital or anorectal (Rectal swab)	Yes	C:ST11:CC11	R	7 (F504L, A510V, I515V, H541N, I566V)	R	493 (WT)	3353 (S821A, K823E, F854L)	2256	2572 (D79N)	22 (-7G deletion)
140808	11.1	South American/UK	2020 strain	H01-GEN-004	SRR36379757	SAMN53825816	2023	20, Male, MSM	Urogenital or anorectal (Rectal swab)	Yes	W:ST11:CC11	R	9 (F504L, A510V, I515V, H541N, I566V)	R	493 (WT)	4108 (K823E, F854L)	1	3151 (mtrR_1:c.337delT (p.Leu114*))	16 (-7G deletion)
140815	11.1	South American/UK	2020 strain	H01-RESP-016	SRR36379743	SAMN53825818	2023	39, Male, Unknown	Respiratory (Throat swab)	Yes	W:ST11:CC11	R	9 (F504L, A510V, I515V, H541N, I566V)	R	493 (WT)	4108 (K823E, F854L)	1	3151 (mtrR_1:c.337delT (p.Leu114*))	16 (-7G deletion)
140817	11.1	South American/UK	2020 strain	H01-RESP-018	SRR36379744	SAMN53825819	2023	28, Male, MSM	Respiratory (Throat swab)	Yes	W:ST11:CC11	R	9 (F504L, A510V, I515V, H541N, I566V)	R	493 (WT)	4108 (K823E, F854L)	1	3151 (mtrR_1:c.337delT (p.Leu114*))	16 (-7G deletion)
140822	11.1	South American/UK	2020 strain	H01-RESP-023	SRR36379745	SAMN53825820	2023	34, Male, MSM	Respiratory (Throat swab)	Yes	W:ST18054:CC11	R	9 (F504L, A510V, I515V, H541N, I566V)	R	493 (WT)	4108 (K823E, F854L)	1	3151 (mtrR_1:c.337delT (p.Leu114*))	16 (-7G deletion)
140825	11.1	South American/UK	2020 strain	H01-RESP-026	SRR36379746	SAMN53825821	2023	29, Male, MSM	Respiratory (Throat swab)	Yes	NG:ST11:CC11	R	9 (F504L, A510V, I515V, H541N, I566V)	R	493 (WT)	4108 (K823E, F854L)	1	3151 (mtrR_1:c.337delT (p.Leu114*))	16 (-7G deletion)
140827	11.1	South American/UK	2020 strain	H01-RESP-028	SRR36379747	SAMN53825822	2023	35, Male, MSM	Respiratory (Throat swab)	Yes	NG:ST11:CC11	S	9 (F504L, A510V, I515V, H541N, I566V)	S	2948 (mtrC_1:c.344_345delCA (p.Ser146*))	4108 (K823E, F854L)	1	3151 (mtrR_1:c.337delT (p.Leu114*))	16 (-7G deletion)
140832	11.1	South American/UK	2020 strain	H01-RESP-033	SRR36379748	SAMN53825823	2023	33, Male, MSM	Respiratory (Throat swab)	Yes	W:ST11:CC11	R	9 (F504L, A510V, I515V, H541N, I566V)	R	493 (WT)	4108 (K823E, F854L)	1	3151 (mtrR_1:c.337delT (p.Leu114*))	16 (-7G deletion)
140850	11.1	South American/UK	South American	H15-RESP-020	SRR36394167	SAMN53856168	2023	46, Male, Unknown	Respiratory (Sputum)	No	W:ST11:CC11	R	952 (F504L, A510V, I515V, H541N, I566V)	S	1 (WT)	4785 (WT)	1	1 (WT)	5 (WT)
140852	11.2	NA	NA	H15-EMI-041	SRR36394171	SAMN53856164	2023	50, Female, NA	Invasive (Blood)	No	C:ST11:CC11	S	7 (F504L, A510V, I515V, H541N, I566V)	S	147 (WT)	257 (WT)	2	1 (WT)	5 (WT)
144661	11.1	South American/UK	2013 strain	H06-EMI-002	SRR36394209	SAMN53856130	2023	18, Female, NA	Invasive (Blood)	No	W:ST11:CC11	S	1 (WT)	S	1 (WT)	1 (WT)	1	989 (WT)	5 (WT)
148039	11.1	South American/UK	2020 strain	H01-RESP-037	SRR36379803	SAMN53825758	2023	35, Male, MSM	Respiratory (Throat swab)	Yes	W:ST18054:CC11	R	1216 (F504L, A510V, I515V, H541N, I566V)	R	493 (WT)	4108 (K823E, F854L)	1	3151 (mtrR_1:c.337delT (p.Leu114*))	16 (-7G deletion)
148040	11.1	South American/UK	2020 strain	H01-RESP-038	SRR36379792	SAMN53825759	2023	37, Male, MSM	Respiratory (Throat swab)	Yes	W:ST18054:CC11	R	1216 (F504L, A510V, I515V, H541N, I566V)	R	493 (WT)	4108 (K823E, F854L)	1	3151 (mtrR_1:c.337delT (p.Leu114*))	16 (-7G deletion)

148046	11.1	South American/UK	2020 strain	H01-RESP-045	SRR36379781	SAMN53825760	2023	31, Male, MSM	Respiratory (Throat swab)	Yes	W:ST11:CC11	S	9 (F504L, A510V, I515V, H541N, I566V)	S	2948 (mtrC_1:c.344_345delCA (p.Ser146*))	4108 (K823E, F854L)	1	3151 (mtrR_1:c.337delT (p.Leu114*))	16 (-7G deletion)
148049	11.1	South American/UK	2020 strain	H01-RESP-048	SRR36379770	SAMN53825761	2023	32, Male, MSM	Respiratory (Throat swab)	Yes	W:ST11:CC11	R	9 (F504L, A510V, I515V, H541N, I566V)	R	493 (WT)	4108 (K823E, F854L)	1	3151 (mtrR_1:c.337delT (p.Leu114*))	16 (-7G deletion)
148053	11.1	South American/UK	2020 strain	H01-RESP-052	SRR36379759	SAMN53825762	2023	36, Male, MSM	Respiratory (Throat swab)	Yes	W:ST11:CC11	R	9 (F504L, A510V, I515V, H541N, I566V)	R	493 (WT)	4108 (K823E, F854L)	1	3151 (mtrR_1:c.337delT (p.Leu114*))	16 (-7G deletion)
148055	11.1	South American/UK	2020 strain	H01-RESP-054	SRR36379741	SAMN53825763	2023	35, Male, MSM	Respiratory (Throat swab)	Yes	W:ST11:CC11	R	9 (F504L, A510V, I515V, H541N, I566V)	R	493 (WT)	4108 (K823E, F854L)	1	3151 (mtrR_1:c.337delT (p.Leu114*))	16 (-7G deletion)
148060	11.1	South American/UK	2020 strain	H01-RESP-060	SRR36379750	SAMN53825765	2023	36, Male, MSM	Respiratory (Throat swab)	Yes	W:ST11:CC11	R	9 (F504L, A510V, I515V, H541N, I566V)	R	493 (WT)	4108 (K823E, F854L)	1	3151 (mtrR_1:c.337delT (p.Leu114*))	16 (-7G deletion)
148078	11.1	South American/UK	2020 strain	H07-RESP-034	SRR36379797	SAMN53825772	2024	50, Male, MSM	Respiratory (Throat swab)	Yes	W:ST11:CC11	R	9 (F504L, A510V, I515V, H541N, I566V)	R	493 (WT)	4108 (K823E, F854L)	1	3151 (mtrR_1:c.337delT (p.Leu114*))	16 (-7G deletion)
148087	11.2	NA	NA	H15-EMI-052	SRR36379788	SAMN53825780	2024	75, Female, NA	Invasive (Blood)	No	C:ST2942:CC11	R	7 (F504L, A510V, I515V, H541N, I566V)	R	493 (WT)	3353 (S821A, K823E, F854L)	2256	2572 (D79N)	22 (-7G deletion)
150022	11.1	South American/UK	2020 strain	H01-RESP-100	SRR36394208	SAMN53856104	2024	28, Male, MSM	Respiratory (Throat swab)	Yes	W:ST11:CC11	R	9 (F504L, A510V, I515V, H541N, I566V)	R	493 (WT)	4108 (K823E, F854L)	1	3151 (mtrR_1:c.337delT (p.Leu114*))	16 (-7G deletion)
150027	11.1	South American/UK	2020 strain	H01-RESP-105	SRR36394197	SAMN53856105	2024	56, Male, MSM	Respiratory (Throat swab)	Yes	W:ST11:CC11	R	9 (F504L, A510V, I515V, H541N, I566V)	R	493 (WT)	4108 (K823E, F854L)	1	3151 (mtrR_1:c.337delT (p.Leu114*))	16 (-7G deletion)
150030	11.1	South American/UK	UK strain	H01-RESP-109	SRR36394186	SAMN53856106	2024	24, Female, NA	Respiratory (Throat swab)	Yes	W:ST11:CC11	S	1 (WT)	S	1 (WT)	1 (WT)	1	1 (WT)	5 (WT)
150588	11.1	South American/UK	2020 strain	H01-EMI-003	SRR36379804	SAMN53825757	2024	64, Male, Unknown	Invasive (Pacemaker)	No	W:ST11:CC11	R	9 (F504L, A510V, I515V, H541N, I566V)	R	493 (WT)	4108 (K823E, F854L)	1	3151 (mtrR_1:c.337delT (p.Leu114*))	16 (-7G deletion)
151639	11.2	NA	NA	H01-RESP-064	SRR36394243	SAMN53856101	2023	25, Male, MSM	Respiratory (Throat swab)	Yes	C:ST11:CC11	R	7 (F504L, A510V, I515V, H541N, I566V)	R	1891 (WT)	5678 (S821A, K823E, F854L)	2	2699 (D79N)	16 (-7G deletion)
151640	11.1	South American/UK	2020 strain	H01-RESP-065	SRR36394242	SAMN53856102	2023	25, Male, MSM	Respiratory (Throat swab)	Yes	W:ST11:CC11	R	9 (F504L, A510V, I515V, H541N, I566V)	R	93 (WT)	5624 (K823E, F854L)	2477	211 (WT)	5 (WT)
151642	11.1	South American/UK	2020 strain	H01-RESP-067	SRR36394219	SAMN53856103	2023	26, Male, MSM	Respiratory (Throat swab)	Yes	W:ST18054:CC11	R	9 (F504L, A510V, I515V, H541N, I566V)	R	493 (WT)	4108 (K823E, F854L)	1	3151 (mtrR_1:c.337delT (p.Leu114*))	16 (-7G deletion)
151661	11.1	South American/UK	2020 strain	H01-RESP-116	SRR36394175	SAMN53856107	2024	34, Male, MSM	Respiratory (Throat swab)	Yes	W:ST11:CC11	R	9 (F504L, A510V, I515V, H541N, I566V)	R	493 (WT)	4108 (K823E, F854L)	1	3151 (mtrR_1:c.337delT (p.Leu114*))	16 (-7G deletion)
151678	11.1	South American/UK	2020 strain	H15-EMI-056	SRR36394170	SAMN53856165	2024	67, Male, Unknown	Invasive (Blood)	No	W:ST18054:CC11	R	9 (F504L, A510V, I515V, H541N, I566V)	R	1565 (WT)	5630 (K823E, F854L)	1	2415 (WT)	22 (-7G deletion)
153697	11.1	South American/UK	2020 strain	H01-RESP-126	SRR36394164	SAMN53856108	2024	26, Male, MSM	Respiratory (Throat swab)	Yes	W:ST11:CC11	R	9 (F504L, A510V, I515V, H541N, I566V)	R	493 (WT)	4108 (K823E, F854L)	1	3151 (mtrR_1:c.337delT (p.Leu114*))	16 (-7G deletion)
153698	11.1	South American/UK	2020 strain	H01-RESP-127	SRR36394153	SAMN53856109	2024	46, Male, MSM	Respiratory (Throat swab)	Yes	W:ST11:CC11	R	9 (F504L, A510V, I515V, H541N, I566V)	R	493 (WT)	4108 (K823E, F854L)	1	3151 (mtrR_1:c.337delT (p.Leu114*))	16 (-7G deletion)
153699	11.1	South American/UK	2020 strain	H01-RESP-128	SRR36394142	SAMN53856110	2024	28, Male, MSM	Respiratory (Throat swab)	Yes	W:ST11:CC11	R	9 (F504L, A510V, I515V, H541N, I566V)	R	493 (WT)	4108 (K823E, F854L)	1	3151 (mtrR_1:c.337delT (p.Leu114*))	16 (-7G deletion)
153707	11.1	South American/UK	2020 strain	H01-RESP-137	SRR36394241	SAMN53856111	2024	38, Male, MSM	Respiratory (Throat swab)	Yes	W:ST11:CC11	R	20 (F504L, A510V, I515V, H541N, I566V)	R	493 (WT)	4108 (K823E, F854L)	1	3151 (mtrR_1:c.337delT (p.Leu114*))	16 (-7G deletion)
153714	11.1	South American/UK	2020 strain	H01-RESP-144	SRR36394230	SAMN53856112	2024	56, Male, MSM	Respiratory (Throat swab)	Yes	W:ST11:CC11	R	9 (F504L, A510V, I515V, H541N, I566V)	R	493 (WT)	4108 (K823E, F854L)	1	3151 (mtrR_1:c.337delT (p.Leu114*))	16 (-7G deletion)
153722	11.1	South American/UK	2020 strain	H07-RESP-038	SRR36394206	SAMN53856132	2024	39, Male, MSM	Respiratory (Throat swab)	Yes	W:ST18054:CC11	R	271 (F504L, A510V, I515V, H541N, I566V)	R	493 (WT)	4108 (K823E, F854L)	1	3151 (mtrR_1:c.337delT (p.Leu114*))	16 (-7G deletion)
154829	11.1	South American/UK	2020 strain	H01-RESP-059	SRR36379749	SAMN53825764	2023	41, Male, MSM	Respiratory (Throat swab)	Yes	W:ST11:CC11	R	9 (F504L, A510V, I515V, H541N, I566V)	R	493 (WT)	4108 (K823E, F854L)	1	3151 (mtrR_1:c.337delT (p.Leu114*))	16 (-7G deletion)
154838	11.1	South American/UK	2020 strain	H01-RESP-153	SRR36379751	SAMN53825766	2024	26, Male, MSM	Respiratory (Throat swab)	Yes	W:ST11:CC11	R	12 (F504L, A510V, I515V, H541N, I566V)	R	493 (WT)	4108 (K823E, F854L)	1	3151 (mtrR_1:c.337delT (p.Leu114*))	16 (-7G deletion)
154845	11.1	South American/UK	2013 strain	H03-RESP-026	SRR36379798	SAMN53825771	2023	88, Female, NA	Respiratory (Sputum)	No	NG:ST11:CC11	R	14 (F504L, A510V, I515V, H541N, I566V)	S	1 (WT)	1 (WT)	1	1 (WT)	5 (WT)
154854	11.1	South American/UK	2020 strain	H14-GEN-001	SRR36379795	SAMN53825774	2024	25, Male, MSM	Urogenital or anorectal (Urine)	Yes	W:ST11:CC11	R	9 (F504L, A510V, I515V, H541N, I566V)	R	493 (WT)	4108 (K823E, F854L)	1	3151 (mtrR_1:c.337delT (p.Leu114*))	16 (-7G deletion)
154855	11.1	South American/UK	2020 strain	H14-GEN-002	SRR36379794	SAMN53825775	2024	25, Male, MSM	Urogenital or anorectal (Rectal swab)	Yes	W:ST11:CC11	R	9 (F504L, A510V, I515V, H541N, I566V)	R	493 (WT)	4108 (K823E, F854L)	1	3151 (mtrR_1:c.337delT (p.Leu114*))	16 (-7G deletion)
154856	11.1	South American/UK	2020 strain	H14-GEN-003	SRR36379793	SAMN53825776	2024	56, Male, MSM	Urogenital or anorectal (Rectal swab)	Yes	W:ST11:CC11	R	9 (F504L, A510V, I515V, H541N, I566V)	R	493 (WT)	4108 (K823E, F854L)	1	3151 (mtrR_1:c.337delT (p.Leu114*))	16 (-7G deletion)
154858	11.1	South American/UK	2020 strain	H14-RESP-032	SRR36379791	SAMN53825777	2024	33, Male, MSM	Respiratory (Throat swab)	Yes	W:ST11:CC11	R	9 (F504L, A510V, I515V, H541N, I566V)	R	493 (WT)	4108 (K823E, F854L)	1	3151 (mtrR_1:c.337delT (p.Leu114*))	16 (-7G deletion)
154863	11.1	South American/UK	2020 strain	H14-RESP-045	SRR36379790	SAMN53825778	2024	29, Male, MSM	Respiratory (Throat swab)	Yes	W:ST11:CC11	R	9 (F504L, A510V, I515V, H541N, I566V)	R	493 (WT)	4108 (K823E, F854L)	1	3151 (mtrR_1:c.337delT (p.Leu114*))	16 (-7G deletion)
154867	11.1	South American/UK	2020 strain	H14-RESP-052	SRR36379789	SAMN53825779	2023	29, Male, MSM	Respiratory (Throat swab)	Yes	W:ST11:CC11	R	9 (F504L, A510V, I515V, H541N, I566V)	R	2510 (WT)	4108 (K823E, F854L)	1	3149 (mtrR_1:c.188dup (p.Glu182*))	16 (-7G deletion)

164980	11.1	South American/UK	2020 strain	H01-RESP-181	SRR36394224	SAMN53856116	2024	26, Male, MSM	Respiratory (Throat swab)	Yes	W:ST11:CC11	S	9 (F504L, A510V, I515V, H541N, I566V)	S	2948 (mtrC_1:c.344_345delCA (p.Ser146*))	4108 (K823E, F854L)	1	3151 (mtrR_1:c.337delT (p.Leu114*))	16 (-7G deletion)
164983	11.1	South American/UK	2020 strain	H01-RESP-184	SRR36394223	SAMN53856117	2024	31, Male, MSM	Respiratory (Throat swab)	Yes	NG:ST11:CC11	S	9 (F504L, A510V, I515V, H541N, I566V)	S	2948 (mtrC_1:c.344_345delCA (p.Ser146*))	4108 (K823E, F854L)	1	3151 (mtrR_1:c.337delT (p.Leu114*))	16 (-7G deletion)
164987	11.1	South American/UK	2020 strain	H01-RESP-188	SRR36394222	SAMN53856118	2024	20, Male, MSM	Respiratory (Throat swab)	Yes	W:ST11:CC11	R	9 (F504L, A510V, I515V, H541N, I566V)	R	493 (WT)	4108 (K823E, F854L)	1	3151 (mtrR_1:c.337delT (p.Leu114*))	16 (-7G deletion)
164990	11.1	South American/UK	2020 strain	H01-RESP-191	SRR36394221	SAMN53856119	2024	32, Male, MSM	Respiratory (Throat swab)	Yes	W:ST11:CC11	R	9 (F504L, A510V, I515V, H541N, I566V)	R	493 (WT)	4108 (K823E, F854L)	1	3151 (mtrR_1:c.337delT (p.Leu114*))	16 (-7G deletion)
164994	11.1	South American/UK	2020 strain	H01-RESP-195	SRR36394220	SAMN53856120	2024	39, Male, MSM	Respiratory (Throat swab)	Yes	W:ST11:CC11	R	9 (F504L, A510V, I515V, H541N, I566V)	R	493 (WT)	4108 (K823E, F854L)	1	3151 (mtrR_1:c.337delT (p.Leu114*))	16 (-7G deletion)
164999	11.1	South American/UK	2020 strain	H01-RESP-199	SRR36394218	SAMN53856121	2024	50, Male, MSM	Respiratory (Throat swab)	Yes	W:ST11:CC11	R	9 (F504L, A510V, I515V, H541N, I566V)	R	493 (WT)	4108 (K823E, F854L)	1	3151 (mtrR_1:c.337delT (p.Leu114*))	16 (-7G deletion)
166442	11.1	South American/UK	2020 strain	H01-RESP-218	SRR36379802	SAMN53825767	2024	21, Male, Unknown	Respiratory (Throat swab)	Yes	W:ST11:CC11	R	9 (F504L, A510V, I515V, H541N, I566V)	R	493 (WT)	4108 (K823E, F854L)	1	3151 (mtrR_1:c.337delT (p.Leu114*))	16 (-7G deletion)
166455	11.2	NA	NA	H01-RESP-231	SRR36379801	SAMN53825768	2024	22, Male, MSM	Respiratory (Throat swab)	Yes	C:ST11:CC11	R	7 (F504L, A510V, I515V, H541N, I566V)	R	493 (WT)	3353 (S821A, K823E, F854L)	2256	2572 (D79N)	22 (-7G deletion)
166483	11.1	South American/UK	2015 strain	H11-EMI-006	SRR36379796	SAMN53825773	2024	85, Male, Unknown	Invasive (Blood)	No	W:ST11:CC11	R	9 (F504L, A510V, I515V, H541N, I566V)	S	2 (WT)	77 (F854L)	1	8 (WT)	5 (WT)
166498	11.1	South American/UK	2020 strain	H15-EMI-063	SRR36379787	SAMN53825781	2025	83, Male, Unknown	Invasive (Blood)	No	W:ST11:CC11	R	9 (F504L, A510V, I515V, H541N, I566V)	R	493 (WT)	4108 (K823E, F854L)	1	3151 (mtrR_1:c.337delT (p.Leu114*))	16 (-7G deletion)
166511	11.1	South American/UK	2020 strain	H15-EMI-062	SRR36394169	SAMN53856166	2024	63, Male, Unknown	Invasive (Unknown)	No	W:ST11:CC11	Unknown	9 (F504L, A510V, I515V, H541N, I566V)	Unknown	493 (WT)	4108 (K823E, F854L)	1	3151 (mtrR_1:c.337delT (p.Leu114*))	16 (-7G deletion)
166607	11.1	South American/UK	2020 strain	H01-RESP-168	SRR36394226	SAMN53856114	2024	26, Male, MSM	Respiratory (Throat swab)	Yes	W:ST17612:CC11	R	9 (F504L, A510V, I515V, H541N, I566V)	R	493 (WT)	4108 (K823E, F854L)	1	3151 (mtrR_1:c.337delT (p.Leu114*))	16 (-7G deletion)
166613	11.1	South American/UK	2020 strain	H01-RESP-174	SRR36394225	SAMN53856115	2024	35, Male, MSM	Respiratory (Throat swab)	Yes	W:ST18054:CC11	R	9 (F504L, A510V, I515V, H541N, I566V)	R	493 (WT)	4108 (K823E, F854L)	1	3151 (mtrR_1:c.337delT (p.Leu114*))	16 (-7G deletion)
166617	11.1	South American/UK	UK strain	H10-EMI-018	SRR36394194	SAMN53856143	2023	23, Female, NA	Invasive (Blood and CSF)	No	W:ST17488:CC11	S	1 (WT)	S	1 (WT)	1 (WT)	1	1 (WT)	5 (WT)
166621	11.1	South American/UK	UK strain	H10-GEN-004	SRR36394193	SAMN53856144	2023	23, Male, Unknown	Urogenital or anorectal (Urethral swab)	Yes	W:ST11:CC11	S	1 (WT)	S	1 (WT)	1 (WT)	1	1 (WT)	5 (WT)
166628	11.1	South American/UK	UK strain	H10-RESP-019	SRR36394192	SAMN53856145	2023	12, Female, NA	Respiratory (Throat swab)	No	NG:ST11:CC11	S	1 (WT)	S	1 (WT)	1 (WT)	1	1 (WT)	5 (WT)
166800	11.1	South American/UK	2020 strain	H01-RESP-241	SRR36379800	SAMN53825769	2024	44, Male, MSM	Respiratory (Throat swab)	Yes	W:ST11:CC11	S	9 (F504L, A510V, I515V, H541N, I566V)	S	2948 (mtrC_1:c.344_345delCA (p.Ser146*))	4108 (K823E, F854L)	1	3151 (mtrR_1:c.337delT (p.Leu114*))	16 (-7G deletion)
167226	11.1	South American/UK	2020 strain	H15-EMI-072	SRR36379786	SAMN53825782	2025	91, Female, NA	Invasive (Blood)	No	W:ST11:CC11	R	11 (F504L, A510V, I515V, H541N, I566V)	R	493 (WT)	4108 (K823E, F854L)	1	3151 (mtrR_1:c.337delT (p.Leu114*))	16 (-7G deletion)
167227	11.1	South American/UK	2020 strain	H15-EMI-075	SRR36379785	SAMN53825783	2025	85, Male, Unknown	Invasive (Blood)	No	W:ST18054:CC11	R	9 (F504L, A510V, I515V, H541N, I566V)	R	493 (WT)	4108 (K823E, F854L)	1	3151 (mtrR_1:c.337delT (p.Leu114*))	16 (-7G deletion)
170420	11.1	South American/UK	2020 strain	H01-EMI-009	SRR36379738	SAMN53825813	2025	53, Male, Unknown	Invasive (Blood)	No	W:ST11:CC11	R	9 (F504L, A510V, I515V, H541N, I566V)	R	493 (WT)	4108 (K823E, F854L)	1	3151 (mtrR_1:c.337delT (p.Leu114*))	16 (-7G deletion)
170422	11.1	South American/UK	2020 strain	H01-RESP-158	SRR36394227	SAMN53856113	2024	24, Male, MSM	Respiratory (Throat swab)	Yes	W:ST11:CC11	R	9 (F504L, A510V, I515V, H541N, I566V)	R	493 (WT)	4108 (K823E, F854L)	1	3151 (mtrR_1:c.337delT (p.Leu114*))	16 (-7G deletion)
170462	11.1	South American/UK	2020 strain	H01-RESP-286	SRR36394217	SAMN53856122	2025	36, Male, MSM	Respiratory (Throat swab)	Yes	W:ST11:CC11	R	9 (F504L, A510V, I515V, H541N, I566V)	R	493 (WT)	4108 (K823E, F854L)	1	3151 (mtrR_1:c.337delT (p.Leu114*))	16 (-7G deletion)
170482	11.1	South American/UK	2020 strain	H01-RESP-308	SRR36394216	SAMN53856123	2025	22, Male, MSM	Respiratory (Throat swab)	Yes	W:ST11:CC11	R	9 (F504L, A510V, I515V, H541N, I566V)	R	493 (WT)	6258 (K823E, F854L)	1	3151 (mtrR_1:c.337delT (p.Leu114*))	16 (-7G deletion)
170506	11.1	South American/UK	2020 strain	H01-RESP-338	SRR36394215	SAMN53856124	2025	36, Male, MSM	Respiratory (Throat swab)	Yes	W:ST11:CC11	R	9 (F504L, A510V, I515V, H541N, I566V)	R	493 (WT)	4108 (K823E, F854L)	1	3151 (mtrR_1:c.337delT (p.Leu114*))	16 (-7G deletion)
170513	11.1	South American/UK	2020 strain	H01-RESP-346	SRR36394214	SAMN53856125	2025	31, Male, MSM	Respiratory (Throat swab)	Yes	W:ST11:CC11	R	9 (F504L, A510V, I515V, H541N, I566V)	R	493 (WT)	4108 (K823E, F854L)	1	3151 (mtrR_1:c.337delT (p.Leu114*))	16 (-7G deletion)
170517	11.1	South American/UK	2020 strain	H02-EMI-012	SRR36379799	SAMN53825770	2025	80, Male, Unknown	Invasive (Blood)	No	W:ST18054:CC11	R	9 (F504L, A510V, I515V, H541N, I566V)	R	493 (WT)	4108 (K823E, F854L)	1	3151 (mtrR_1:c.337delT (p.Leu114*))	16 (-7G deletion)
170536	11.1	South American/UK	2020 strain	H10-RESP-050	SRR36394191	SAMN53856146	2025	66, Male, Unknown	Respiratory (Sputum)	No	W:ST11:CC11	R	9 (F504L, A510V, I515V, H541N, I566V)	R	493 (WT)	4108 (K823E, F854L)	1	3151 (mtrR_1:c.337delT (p.Leu114*))	16 (-7G deletion)
170541	11.1	South American/UK	2020 strain	H14-RESP-044	SRR36394180	SAMN53856156	2023	36, Male, MSM	Respiratory (Throat swab)	Yes	W:ST11:CC11	R	9 (F504L, A510V, I515V, H541N, I566V)	R	493 (WT)	4108 (K823E, F854L)	1	3151 (mtrR_1:c.337delT (p.Leu114*))	16 (-7G deletion)
170548	11.1	South American/UK	2013 strain	H15-EMI-077	SRR36394168	SAMN53856167	2025	51, Female, NA	Invasive (Blood and CSF)	No	W:ST11:CC11	S	1 (WT)	S	1 (WT)	1 (WT)	1	989 (WT)	5 (WT)

1. Sexual orientation data was only collected for male individuals.

STI: sexually transmitted infection; ST: sequence-type; CC: clonal complex; UK: United Kingdom; NA: not applicable; R: resistant; S: susceptible; WT: wild-type

Supplementary Table 2. List of *Neisseria meningitidis* genomes belonging to clonal complex 11 used as reference strains in this study.

PubMLST ID	Lineage	Sublineage	Genetic variant	Run Accession	Country	Year	SG	ST	CC	Belonging
71709	Lineage 11.2	-	-	SRR10610845	New Zeland	2018	C	11	11	https://doi.org/10.1016/j.jinf.2015.07.007
93928	Lineage 11.1	-	Proximal region	ERR3606580	Ireland	2018	C	11	11	
29709	Lineage 11.1	-	Intermediate region	ERR558095	UK	2007	W	247	11	https://doi.org/10.1016/j.jinf.2015.07.007
2290	Lineage 11.1	African/Hajj outbreak	Hajj outbreak	SRR513875	Saudi Arabia	2000	W	11	11	https://doi.org/10.1086/340414<br>https://doi.org/10.1016/j.ebiom.2015.09.007
30106	Lineage 11.1	African/Hajj outbreak	Burkina Faso	ERR557770	Burkina Faso	2001	W	11	11	https://doi.org/10.1016/j.jinf.2015.07.007
29355	Lineage 11.1	African/Hajj outbreak	Endemic South Africa	ERR519856	South Africa	2009	W	11	11	https://doi.org/10.1016/j.jinf.2015.07.007
31151	Lineage 11.1	South American/UK	South American	ERR1124363	Argentina	2011	W	10857	11	https://doi.org/10.1016/j.jinf.2015.07.007
31156	Lineage 11.1	South American/UK	South American	ERR1124366	Brazil	2011	W	11	11	https://doi.org/10.1016/j.jinf.2015.07.007
30154	Lineage 11.1	South American/UK	Original UK	ERR557579	United Kingdom	2014	W	11	11	https://doi.org/10.1016/j.jinf.2015.07.007
30167	Lineage 11.1	South American/UK	2013 strain	ERR557603	United Kingdom	2014	W	11	11	https://doi.org/10.1016/j.jinf.2015.07.007
46309	Lineage 11.1	South American/UK	2015 strain	SRR30268310	Australia	2016	W	11	11	https://doi.org/10.3201/eid2308.170259
71724	Lineage 11.1	South American/UK	2015 strain	SRR10610828	New Zeland	2016	W	11	11	https://doi.org/10.3201/eid2704.191716

SG: serogroup; ST: sequence-type; CC: clonal complex.

Supplementary Table 3. Classification of *Neisseria meningitidis* isolates belonging to clonal complex 11 according to sampling source and genogroup.

	**Invasive isolates (n=108) n (%)**	**Respiratory isolates obtained during STI screening (n=53) n (%)**	**Respiratory isolates (n=10) n (%)**	**Urogenital/anorectal isolates (n=8) n (%)**	**Total (n=179) n (%)**
Genogroup W	75 (69.4)	47 (88.6)	4 (40.0)	6 (75.0)	132 (73.7)
Genogroup C	31 (28.7)	3 (5.7)	1 (10.0)	1 (12.5)	36 (20.1)
Genogroup B	2 (1.9)	0	0	0	2 (1.1)
Non-groupable	0	3 (5.7)	5 (50.0)	1 (12.5)	9 (5.0)
